# Web-Based Gamified Auditory-Cognitive Dual-Task Training for Older Adults With Age-Related Hearing Loss: Pilot Randomized Controlled Trial

**DOI:** 10.2196/84083

**Published:** 2026-06-16

**Authors:** Ivy Yan Zhao, Angela Yee Man Leung, Chen Li, Laurence Lloyd Parial, Hongming Ma, Jed Montayre, Justin S Golub, Robert Sweetow, Janet Ho-Yee Ng, Engle Angela Chan

**Affiliations:** 1 School of Nursing, The Hong Kong Polytechnic University, 11 Yuk Choi Road, Hung Hom, Hong Kong, China (Hong Kong), 852 27664550; 2 WHO Collaborating Centre for Community Health Services, School of Nursing, The Hong Kong Polytechnic University, Hong Kong, China (Hong Kong); 3 Research Institute for Smart Ageing (RISA), The Hong Kong Polytechnic University, Hong Kong, China (Hong Kong); 4 Department of Applied Social Sciences & Department of Computing, The Hong Kong Polytechnic University, Hong Kong, China (Hong Kong); 5 College of Nursing, University of the Philippines Manila, Manila, National Capital Region, Philippines; 6 JBI Hong Kong Centre of Evidence-based Healthcare Excellence, School of Nursing, The Hong Kong Polytechnic University, Hong Kong, China (Hong Kong); 7 Department of Otolaryngology-Head and Neck Surgery, Columbia University Vagelos College of Physicians and Surgeons, NewYork-Presbyterian/Columbia University Irving Medical Center, New York, NY, United States; 8 School of Medicine, University of California, San Francisco, San Francisco, CA, United States; 9 Department of Language Science and Technology, The Hong Kong Polytechnic University, Hong Kong, China (Hong Kong)

**Keywords:** randomized controlled trial, age-related hearing loss, cognitive training, auditory training, gamified, dual-task training

## Abstract

**Background:**

Everyday listening ability is essential for individual health and well-being. Age-related hearing loss (ARHL) is associated with reduced communication engagement, social isolation, loneliness, cognitive decline, and increased dementia risk. Interventions that simultaneously target auditory processing and cognitive function, particularly within engaging and ecologically valid contexts, may offer greater benefits than unimodal approaches. However, culturally adapted, web-based, gamified auditory-cognitive dual-task training (ACDT) tailored for older adults with ARHL remains underexplored. At the time of this writing, few auditory or auditory-cognitive training programs are available in Chinese languages, creating linguistic and cultural barriers for older adults.

**Objective:**

This study aimed to (1) assess the feasibility and acceptability of home-based ACDT among older Chinese adults with ARHL and (2) examine its preliminary effects on global cognition, hearing, social engagement, and loneliness. It was hypothesized that the intervention group would demonstrate greater improvements in global cognition, hearing, and social engagement than the control group.

**Methods:**

Sixty community-dwelling older adults with mild-to-moderate ARHL were randomized 1:1 to either the ACDT group or a waitlist control group in a single-blinded pilot randomized controlled trial. Demographic data and outcome measures were collected at baseline, week 6, and week 12. Postintervention interviews were conducted to assess the feasibility and acceptability of ACDT.

**Results:**

A total of 60 participants were randomized (mean age 67.65, SD 4.78 years; 45/60, 75% male). ACDT demonstrated high feasibility and acceptability. The ACDT group showed significant improvements in focused attention (mean change=–0.15; *P=.*02; *d*=–0.46) and divided attention (mean change=–0.21; *P=.*002; *d*=–0.63). Significant cognitive improvements on the Hong Kong-Montreal Cognitive Assessment were identified in naming (*r*=0.37; *P=.*05) and visual cognition (*r*=0.44; *P=.*02) in the intervention group, while no significant improvements were found in the control group. Both groups reported significant decreases in emotional hearing handicap, with slightly greater improvement in the intervention group (*r*=0.39; *P=.*03) than in the control group (*r*=0.37; *P=.*04). Linear mixed model analysis revealed a small to moderate group effect (Cohen *d*=0.38) for 5-minute delayed recall on the Auditory Verbal Learning Test, with the fixed effects explaining 69% of the variance (marginal *R²*=0.69). A significant time×group interaction was observed for left-ear thresholds (*P=.*01). Qualitative analysis identified three key themes: (1) intervention coherence and participants’ affective attitude toward ACDT; (2) perceived benefits in cognition, information acquisition, and self-awareness from ACDT; and (3) perceiving ACDT as less burdensome with enhanced self-efficacy.

**Conclusion:**

Future iterations should incorporate artificial intelligence–enhanced personalization. Large-scale randomized controlled trials involving diverse samples and active control conditions are needed to confirm sustained effects on auditory and cognitive health, dual-task listening-cognitive abilities, and real-world functioning.

## Introduction

Everyday listening ability is essential for individual health and well-being. Age-related hearing loss (ARHL) represents a highly prevalent sensory deficit, affecting approximately one-third of adults aged 60 years or older [1,2]. ARHL is associated with reduced communication engagement, social isolation, and loneliness [3-5]. Beyond these social consequences, ARHL shows consistent associations with cognitive decline and increased risk of dementia [6-8]. Research indicates that even subclinical ARHL may increase the long-term risk of cognitive decline and dementia [9-12], with greater hearing loss severity correlating with poorer performance on memory, executive function, and mental status measures [13,14].

The mechanisms underlying these associations appear multifactorial, involving a complex interplay of sensory deprivation, increased cognitive load during auditory processing, accelerated brain atrophy (particularly in temporal regions), and reduced social-cognitive stimulation leading to neural disuse [15-19]. The sensory deprivation hypothesis suggests that prolonged reductions in sensor input (eg, hearing loss) increase the cognitive effort required for listening, and are subsequently associated with neural atrophy [17-19], while reduced auditory stimulation may cause structural brain changes, such as cortical thinning, reduced gray matter volume, and altered connectivity in auditory and multimodal networks [15,16]. The Cattell-Horn-Carroll theory of cognitive abilities provides a structured framework for describing and classifying the cognitive domains examined in relation to ARHL [20]. For example, increased listening effort may place greater demands on cognitive resources, particularly working memory and processing speed. This chronic reallocation of resources may reduce the capacity available for higher-order cognitive functions, including novel problem-solving, memory retrieval, and the application of acquired knowledge. Thus, hearing loss can cascade into broader cognitive impairment, extending beyond auditory processing alone [20]. Longitudinal evidence indicates that untreated ARHL is associated with a 30%‐50% increased risk of incident dementia and accelerates cognitive decline by 30%‐40% over 6‐10 years [13,21,22]. Hearing loss treatment in mid-life may reduce dementia risk by 8% [23] with potential benefits in lowering health care use and costs [24,25]. These findings highlight the need for early intervention to mitigate the cognitive risks associated with ARHL.

While hearing aids may help mitigate the risk of cognitive decline [26], adherence challenges persist, and their benefits might be limited primarily to peripheral hearing deficits [27]. However, ARHL also involves central auditory processing difficulties, which appear to be more strongly associated with cognitive impairment than pure tone thresholds. These central deficits place significant demands on cognitive domains that are essential for understanding speech in complex listening environments. Accordingly, interventions that simultaneously target auditory processing and cognitive function [27], particularly in engaging and ecologically valid contexts, may offer greater benefits than unimodal approaches [28,29]. The auditory trainings for older adults with hearing loss mainly focus on speech or word recognition in noise, with auditory processing as the primary outcome [28]. Some studies have expanded these approaches by combining auditory training with cognitive training [30], contextual and visual cues [31], or elements of real-world communication [32]. For example, a randomized controlled trial (RCT) in the United Kingdom used computer-delivered phoneme training with 11 synthesized phoneme continua derived from natural voice recordings and implemented using a 3-interval, 3-alternative forced-choice oddball paradigm. Although the training produced substantial on-task learning effects in older adults with hearing loss, further research is needed to determine which training tasks are most effective, especially given the considerable individual variability in outcomes [30]. Dual-task training approaches, which require participants to perform distinct measurable tasks concurrently or sequentially [33], represent a promising direction for maximizing cognitive benefits [34]. Such training may improve the allocation of limited cognitive resources under challenging listening conditions while also providing repeated stimulation that could support neuroplastic changes in relevant networks [27]. In parallel, web-based cognitive training has shown promise for enhancing cognition [35,36], and gamification may further improve engagement in digital health interventions [37].

However, culturally adapted, web-based, gamified auditory-cognitive dual-task training (ACDT) tailored for older adults with ARHL remains underexplored. At the time of this writing, few auditory or auditory-cognitive trainings are available in Chinese languages, creating linguistic and cultural barriers for older adults. Given evidence that auditory training delivered in one’s native language may enhance comprehension, engagement, and listening skill transfer [38], this study aimed to (1) assess the feasibility and acceptability of a web-based, gamified ACDT program performed at home among older Chinese adults with ARHL, and (2) examine its preliminary effects on global cognition, hearing, social engagement, and loneliness. It was hypothesized that the intervention group would demonstrate greater improvements in global cognition, hearing, and social engagement compared with the control group.

## Methods

### Study Design

A prospective, single-blind, 2-arm parallel pilot RCT, followed by descriptive semistructured individual interviews, was conducted between April 28, 2023, and June 27, 2025. Reporting followed CONSORT (Consolidated Standards of Reporting Trials; Checklist 1) guidelines [39], with the trial protocol available in Multimedia Appendix 1.

### Ethical Considerations

The institutional review board of the Hong Kong Polytechnic University (HSEARS20230223006) approved the study. All participants enrolled in this study voluntarily and signed informed consent before the formal start of the assessment after being informed that they could withdraw at any time. Each participant was introduced to the aims and content of the study, potential risks, and benefits. All participants provided written informed consent in accordance with the Declaration of Helsinki. HKD $200 (~US $26; a currency exchange rate on April 28, 2023, is applicable) supermarket vouchers were provided to participants as an incentive to compensate for their time spent on the interview and completion of questionnaires. All data, audio recordings, and written records are stored on a password-protected laptop; only the research team has access to them. In addition, all data were used for research purposes only and could not be disclosed without the participants’ consent. To ensure privacy protection, the participants’ personal information was anonymized and labeled “P1-P60.”

### Study Participants

Participants were recruited using convenience sampling through community health centers and advertisements across Hong Kong (eg, social media). Eligibility criteria included (1) aged 60 years or older and living in the community; (2) mild-to-moderate hearing loss, defined as a pure-tone average (PTA) of 20‐50 dB across octave frequencies from 0.5 to 4 kHz in both ears; (3) no prior use of hearing aids; (4) normal cognitive performance (Montreal Cognitive Assessment score ≥26); and (5) willingness and ability to provide informed consent and comply with study procedures. Exclusion criteria were (1) a history of psychosis, mania, bipolar disorder, substance use disorder, or suicidal ideation; (2) severe or unstable medical illness; (3) significant retrocochlear pathology or an organic lesion causing hearing loss; (4) a diagnosis of probable Alzheimer disease, vascular dementia, frontotemporal dementia, or Parkinson disease; and (5) use of medications, such as antidepressants, sedatives, or antiepileptics, that may affect cognition.

### Sample Sizes

This pilot RCT was not designed to definitively test intervention effects, which would require a fully powered trial. Due to the absence of established effect sizes from previous literature, a target sample of 60 participants (30 per arm), allowing for 15% attrition, was determined based on recommendations for pilot RCTs [40,41] and with consideration of the study objectives, timeline, budget, and available tablets.

### Interventions

**Figure 1.**
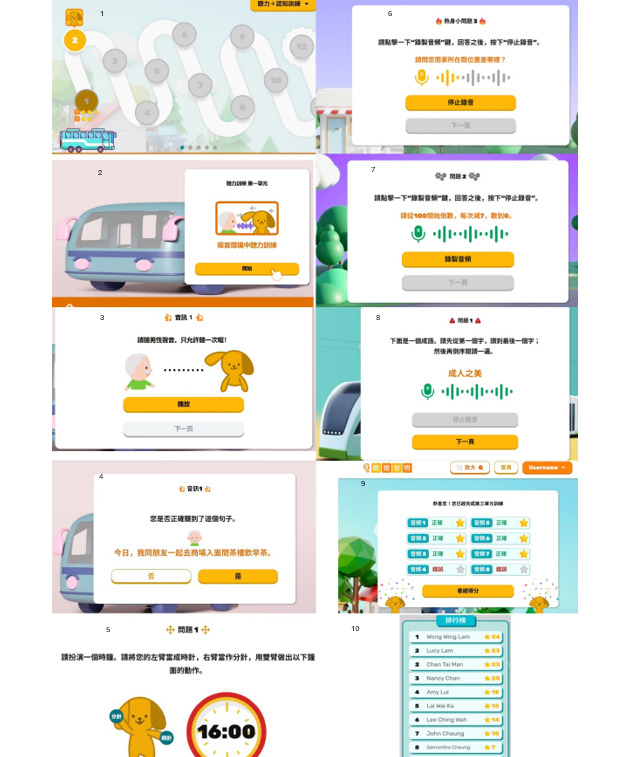
Screenshot examples of the web-based gamified auditory-cognitive dual-task training (ACDT) system developed by the team.

The ACDT training system was co-designed by the research team and older adults with hearing loss, in collaboration with service providers in Hong Kong, and is patented (HK30110757) [42]. The 12-week training intervention consisted of five 60-minute sessions per week [42] and used a sequential dual-task training approach [33]. To enhance engagement, the system incorporates sound effects and interactive gamification elements, including likes, praise, fireworks, and leaderboards (Figure 1). In Figure 1, screenshot 1 illustrates the daily training pathway (only one module is activated per day). Screenshots 2‐4 show a listening task with background noise, in which the participant listens to a sentence spoken by a male voice, repeats it, and checks whether all words were correctly heard. Screenshots 5‐8 demonstrate different cognitive training tasks, including indicating a time using hand gestures, identifying locations, counting numbers, and reading Chinese idioms forward and backward. Screenshot 9 shows the scoring system, and Screenshot 10 presents the ranking list. It also offers participants flexibility for individual training while fostering a sense of virtual group participation through the leaderboard.

Each session begins with a 5-minute volume adjustment to establish a comfortable listening level, followed by attention or orientation training exercises where participants answer questions related to person (identifying their name), time (date, month, or year), and place (location at the time of this writing). This was followed by 25 minutes of auditory training based on culturally adapted exercises from the Listening and Communication Enhancement program [43]. The auditory training includes speech-in-noise perception, rapid speech comprehension, and competing speaker discrimination, all contextualized within real-life scenarios such as medical visits or dim sum gatherings. Difficulty hearing a speaker in noisy environments is a common and frustrating consequence of hearing loss. In the training, participants were trained to listen to a medium-length sentence presented in background noise, repeat it aloud, and then see the sentence in writing to confirm whether they understood every word. The difficulty levels of the sentence and background noise are automatically adjusted based on the participant’s performance on the previous sentence. This training enhances the brain’s auditory processing by helping participants filter out background noise and focus on the target voice. Understanding rapid speech is another common challenge for older adults with hearing loss. In this training, the system automatically adapts the speed of sentences to help participants focus on fast talkers, aiding in comprehension. Additionally, ARHL makes listening difficult when multiple speakers are talking. During the training, participants listen to one of 3 voices (male, female, or child) as the target voice, while a competing voice is presented at varying volumes to create a challenge. This training teaches the participant’s brain to ignore a competing speaker’s voice and focus on the conversation they are engaged in. The auditory training was followed by 25 minutes of cognitive training delivered in a game-based format. These activities target executive function (forward/backward serial counting), perceptual-motor ability (arm-clock positioning), memory (forward/backward repetition of numbers/words), and complex attention (forward/backward spelling of Chinese idioms/poetry) using culturally relevant materials. Sessions concluded with a 5-minute wrap-up featuring communication strategy review (“learning classroom”) and recall of visuospatial and memory tasks.

### Waitlist Control

The waitlist control group received no intervention during the study period but was given access to the ACDT after T2 data collection.

### Randomization

Stratified randomization based on key sociodemographic factors (eg, age, gender, and education level) was used to ensure balanced group allocation. An independent statistician assigned participants to either the ACDT or the waitlist control group in a 1:1 ratio using R software (R Core Team). Group assignments were placed in sealed opaque envelopes, and participants were informed of their allocation after completing all baseline assessments.

### Study Procedures

After obtaining informed consent, a blinded researcher collected demographic data and administered outcome measures at baseline (T0), week 6 (T1), and week 12 immediately postintervention (T2). The week-6 assessment was included to identify potential early intervention effects, monitor participant attrition patterns, and verify adherence to the intervention dosage. Prior to intervention commencement, participants attended briefing sessions on system operation and received personal accounts with instructional videos. Technical support was available via a hotline.

All the ACDT participants were invited to take part in an individual interview at T2 following a semistructured interview guide (Textbox 1). The interviews were informed by the theoretical framework of acceptability (TFA) [44], which includes 7 constructs, including affective attitude, burden, perceived effectiveness, ethicality, intervention coherence, opportunity costs, and self-efficacy [44]. The TFA was used in the present study to gain a comprehensive understanding of participants’ experiences with the ACDT, as well as their perceptions of and acceptability toward the intervention and study design. Interviews were held at community centers or in a university lab and lasted around 45‐60 minutes. All the interviews were audio recorded to facilitate data analysis. Interview data were reviewed regularly by the study team until no new topics emerged, indicating that data saturation had been reached [45].

Textbox 1. **Semistructured interview guide.**

Example of questions

- How was your experience of participating in this study?
- Please tell me about your overall experience of attending the auditory-cognitive dual-task training (ACDT) sessions.
- Have you noted any changes in your health/daily life after participating in the ACDT? If so, do you think these changes were related to the training?
- Which ACDT task did you find most helpful, and which did you find least helpful? Please explain your reasons. What made them useful or not useful? How was your experience with the auditory training in this program?
- How was your experience with the cognitive training in this program?
- How was your experience performing the auditory and cognitive trainings simultaneously?
- What difficulties or problems did you experience when using the program?
- Would you like to continue this training at home in the future?
- What did the instructors or facilitators do that was most/least helpful?
- How do you think about the group-based setup of this ACDT program?
- Do you have any suggestions for improving the ACDT program?

### Outcome Measures

#### Primary Outcome

The primary outcome of global cognition was assessed using a neuropsychological battery comprising (1) the Chinese Auditory Verbal Learning Test (AVLT), which assesses multiple cognitive domains including immediate recall, learning efficiency, delayed recall, retention rate, and recognition memory, and has demonstrated high test-retest reliability (intraclass correlation coefficient [ICC]=0.79‐0.82) and 78% sensitivity; (2) the Chinese Trial Making Test Parts A and B, which measures visual attention, processing speed, and executive function, and has shown strong discriminant validity (Cohen *d*=1.2 for vascular dementia) [46]; and (3) the Hong Kong-Montreal Cognitive Assessment (HK-MoCA), a 30-point cognitive screening tool covering attention, naming, memory, language, visuospatial skills, abstraction, and orientation, with reported sensitivity of 89% and reliability (ICC=0.89‐0.92) [47]. Alternating versions of HK-MoCA were used to minimize practice effects.

#### Secondary Outcomes

Secondary outcomes included: (1) hearing, measured by PTA and the Chinese version of the Hearing Handicap Inventory for the Elderly, which has demonstrated strong internal consistency (α=.91) and convergent validity with PTA (*r*=0.74) [48]; (2) social engagement assessed via the validated 6-item Chinese version of the Lubben Social Network Scale, with high test-retest reliability (ICC=0.85) [49]; and (3) loneliness measured by the 6-item Chinese De Jong Gierveld loneliness scale, demonstrating good internal consistency (α=.80) and discriminative validity for social isolation (area under the curve [AUC]=0.79) [50].

Covariates encompassed age, gender, baseline hearing and cognitive function, duration of hearing loss, education level, health status, and psychological well-being.

#### Feasibility and Acceptability

The feasibility and acceptability of the ACDT were assessed based on qualitative interviews and comprehensive logs documenting recruitment, retention, adherence, participant contacts, implementation challenges, withdrawal reasons, and completion factors (Multimedia Appendix 2). The intervention completion rate was defined as the extent to which participants completed the 60 training modules during the 12-week intervention period. System-generated data (training dates, progress, and performance metrics) were downloaded after the intervention.

### Statistical Analysis

Data analyses and plotting were performed using SPSS Statistics (version 29.0.2.0; IBM Corp) and Python (version 3.11; Python Software Foundation). A 2-sided *P*<.05 was set as the predetermined threshold for statistical significance. Missing data (<7% across variables) were imputed using group means to support analyses requiring complete paired observations and to avoid excluding participants due to missing covariates. A natural log transformation was applied to TMT scores at all time points to address skewness. Baseline group equivalence was confirmed via independent *t* tests (continuous variables) and chi-square tests (categorical variables). The primary analysis focused on within-group changes from baseline to T2. Normality of the distribution of outcome change scores was evaluated using the Shapiro–Wilk test. Based on the results, within-group changes for normally distributed variables (*P*≥.05) were analyzed using paired *t* tests, with Cohen *d* calculated to measure effect size. For nonnormally distributed variables (*P*<.05), the nonparametric Wilcoxon signed-rank test was used.

Effect size *r* for the Wilcoxon signed-rank tests was calculated based on the standardized test statistic (*Z*) and the total number of observations. Effect sizes were interpreted using established criteria, with thresholds of 0.1, 0.3, and 0.5 indicating small, medium, and large effects, respectively. Between-group differences across time points (baseline, wk-6, and wk-12) were evaluated using linear mixed effects models. Each model included time (categorical, 3 levels), group, and the time×group interaction as fixed effects, while adjusting for the baseline value of the respective outcome as a covariate. Within-participant correlations across time points were modeled using a first-order autoregressive residual covariance structure. Models were fitted using restricted maximum likelihood. Pairwise comparisons of estimated marginal means for time×group interactions were conducted using the least significant difference adjustment. Given the explorative nature of the study, no adjustment for multiplicity was applied to the multiple comparisons of global cognition scores.

Qualitative data were analyzed using the hybrid thematic analysis approach described by Braun and Clarke [51], combining inductive coding with deductive theme organization guided by the TFA [44]. Two researchers (IYZ and HM) independently analyzed data in Chinese to preserve the original meaning conveyed by participants. Initial codes were generated directly from the transcripts and then mapped onto TFA domains using NVivo (version 14; Lumivero). Codes that did not align with existing TFA domains were used to inform theme refinement or adaptation. All codes and preliminary themes were documented in a spreadsheet, translated into English, and then back-translated into Chinese by HM and verified by IYZ. Any discrepancies were solved with input from the study team.

## Results

### Participants

A total of 60 participants were randomized. Baseline characteristics were comparable between groups regarding age, gender, education, employment status, marital status, living arrangements, and health status (Table 1). The mean age of the participants was 67.65 years (SD 4.78). Out of 60 participants, the cohort included 45 (75%) men and 15 (25%) women. Most participants (47/60, 78%) were retired and had attained an upper-secondary education or above. Seventy percent were married, while the majority (52/60, 87%) lived with a spouse, family members, or friends. Regarding health status, 42% (25/60) self-reported their health as good to very good, while 52% (31/60) reported their health as normal.

**Table 1.** Demographic characteristics of the participants.

Outcomes	Total (n=60)	Intervention (n=30)	Control (n=30)	Test	*P* value
Age (year), mean (SD)	67.65 (4.78)	67.73 (4.38)	67.57 (5.15)	*t* test^a^	.90
Sex, n (%)				*χ*²^b^	.60
Female	15 (25)	6 (20)	9 (30)		
Male	45 (75)	24 (80)	21 (70)		
Education, n (%)				Fisher exact test	.90
Primary school	2 (3)	1 (3)	1 (3)		
Lower-secondary	9 (15)	3 (10)	6 (20)		
Upper-secondary	26 (43)	14 (47)	12 (40)		
Bachelor’s or equivalent	17 (28)	9 (30)	8 (27)		
Master’s or equivalent	6 (10)	3 (10)	3 (10)		
Employment, n (%)				Fisher exact test	.08
Retired	47 (78)	24 (80)	23 (77)		
Full-time employed	5 (8)	0 (0)	5 (17)		
Part-time employed	4 (7)	3 (10)	1 (3)		
Seeking work	1 (2)	1 (3)	0 (0)		
Housewife	3 (5)	2 (7)	1 (3)		
Marital status, n (%)				Fisher exact test	.22
Married	42 (70)	23 (77)	19 (63)		
Single	14 (23)	4 (13)	10 (33)		
Divorced	1 (2)	1 (3)	0 (0)		
Widowed	3 (5)	2 (7)	1 (3)		
Living arrangement, n (%)				Fisher exact test	.60
Live alone	8 (13)	4 (13)	4 (13)		
With spouse	24 (40)	14 (47)	10 (33)		
With family	27 (45)	12 (40)	15 (50)		
With friend	1 (2)	0 (0)	1 (3)		
Health status, n (%)				Fisher exact test	.57
Not good	4 (7)	3 (10)	1 (3)		
Normal	31 (52)	14 (47)	17 (57)		
Good	24 (40)	12 (40)	12 (40)		
Very good	1 (2)	1 (3)	0 (0)		

a Continuous variable: independent-samples *t* test,

b Categorical variables: chi-square test or Fisher exact test.

### Feasibility of the Intervention

**Figure 2.**
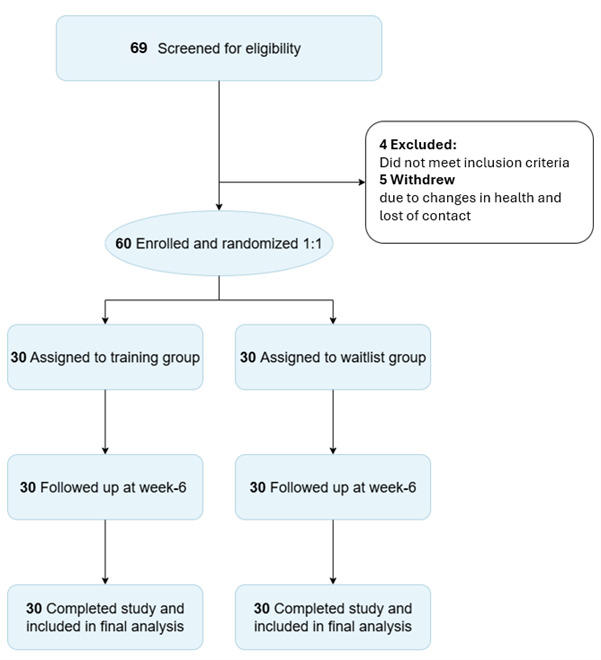
CONSORT (Consolidated Standards of Reporting Trials) diagram.

The trial proceeded according to the protocol without early termination. There were 69 candidates screened for eligibility; 4 were excluded for not meeting the eligibility criteria, and 5 withdrew prior to randomization due to health issues or loss of contact (Figure 2). Of the 60 participants who met the eligibility criteria, 30 were randomized to the ACDT group, and 24 of them completed all 60 training modules, while 6 participants completed 55 modules. The overall intervention completion rate was nearly 98% (1770/1800). At the beginning of the study, our hotline received a small number of calls regarding login issues, primarily from participants who had forgotten their passwords or were unsure how to reset them. This issue was resolved after we improved the study briefing sessions. No adverse events were reported in either the intervention or control group.

### Quantitative Results

Within-group analyses showed significant decreases in TMT completion times over 12 weeks, reflecting improved performance in focused attention (intervention: mean change=–0.15; *P=.*02; *d*=–0.46; control: mean change=–0.10; *P=.*027; *d*=–0.46), and divided attention (intervention: mean change=–0.21; *P=.*002; *d*=–0.63; control: mean change=–0.11; *P=.*02; *d*=–0.45). The intervention group demonstrated moderately greater improvements than the control group (Table 2). Both groups reported a significant decrease in overall hearing handicap. No statistically significant within-group changes were observed in HK-MoCA total scores or in social relationship and loneliness scores in either group.

**Table 2.** Results of a paired *t* test.

Variable and group		Mean change	*P* value	Effect size (Cohen *d*)
Focused attention (TMT-A)^a^				
Intervention		–0.15	.02	–0.46
Control		–0.10	.02	–0.46
Divided attention (TMT-B)^b^				
Intervention		–0.21	.002	–0.63
Control		–0.11	.02	–0.45
HK-MoCA total score^c^				
Intervention		0.40	.30	0.19
Control		–0.13	.74	–0.06
Hearing handicap (HHIE), overall^d^				
Intervention		–2.43	.05	–0.37
Control		–3.83	.005	–0.56
Hearing handicap (HHIE), social^d^				
Intervention		–1.79	.34	–0.42
Control		–2.50	.14	–0.65
Social engagement (Lubben Social Network Scale)				
Intervention		0.60	.43	0.15
Control		1.00	.11	0.30
PTA (left ear)^e^				
Intervention		–1.64	.16	–0.26
Control		–0.42	.63	–0.09
PTA (right ear)^e^				
Intervention		–1.39	.26	–0.21
Control		–1.79	.08	–0.33

a TMT-A: Trial Making Test Part A.

b TMT-B: Trial Making Test Part B,

c HK-MoCA: Hong Kong-Montreal Cognitive Assessment.

d HHIE: Hearing Handicap Inventory for the Elderly.

e PTA: pure-tone average.

For outcome variables that violated the normality assumptions, Wilcoxon signed-rank tests were conducted separately within each group (Table 3). In the intervention group, significant cognitive improvements were observed in the HK-MoCA subdomains of naming (*Z*=2.00; *P=.*05; *r*=0.37) and visual cognition (*Z*=2.39; *P=.*02; *r*=0.44), while the control group showed no significant within-group changes in these areas. Both groups reported significant decreases in emotional hearing handicap; however, the intervention group demonstrated a larger effect size (*Z*=2.15; *P=.*03; *r*=0.39) than the control group (*Z*=2.03; *P=.*04; *r*=0.37). Similarly, both groups demonstrated significant improvements in 5-min delayed recall on the AVLT, with the intervention group exhibiting greater improvement (*Z*=4.16; *P<*.001; *r*=0.76) than the control group (*Z*=4.11; *P<*.001; *r*=0.75).

**Table 3.** Results of Wilcoxon signed-rank tests.

Outcomes and group		Z	*P* value	Effect size (*R*)	Magnitude
Naming (HK-MoCA)^a^					
Intervention		2.00	.05	0.37	Medium
Control		1.63	.10	0.30	Small
Visual cognition (HK-MoCA)					
Intervention		2.39	.02	0.44	Medium
Control		1.15	.25	0.21	Small
Hearing handicap (emotional), HHIE^b^					
Intervention		2.15	.03	0.39	Medium
Control		2.03	.04	0.37	Medium
5-min delayed recall (AVLT^c^)					
Intervention		4.16	<.001	0.76	Large
Control		4.11	<.001	0.75	Large
Recognition (AVLT)^c^					
Intervention		1.81	.07	0.33	Medium
Control		2.08	.04	0.38	Medium
Language (HK-MoCA)					
Intervention		N/A^d^	N/A	N/A	N/A
Control		1.34	.18	0.24	Small
Memory (HK-MoCA)					
Intervention		0.99	>.99	0.18	Small
Control		1.28	.41	0.23	Small
Orientation (HK-MoCA)					
Intervention		1.63	.10	0.30	Small
Control		0.00	>.99	0.00	Trivial
Abstract (HK-MoCA)					
Intervention		0.92	.36	0.17	Small
Control		0.28	.78	0.05	Trivial
Attention (HK-MoCA)					
Intervention		0.83	.41	0.15	Small
Control		0.55	.58	0.10	Small
Loneliness (single question)					
Intervention		0.00	>.99	0.00	Trivial
Control		0.82	.41	0.15	Small
Emotional loneliness					
Intervention		0.68	.50	0.12	Small
Control		0.00	>.99	0.00	Trivial
Social loneliness					
Intervention		0.13	.89	0.02	Trivial
Control		1.22	.22	0.22	Small
Overall loneliness					
Intervention		0.51	.61	0.09	Trivial
Control		0.90	.31	0.16	Small

a HK-MoCA: Hong Kong-Montreal Cognitive Assessment.

b HHIE: Hearing Handicap Inventory for the Elderly.

c AVLT: Chinese Auditory Verbal Learning Test.

d N/A: not applicable due to zero variance in the difference scores.

**Figure 3.**
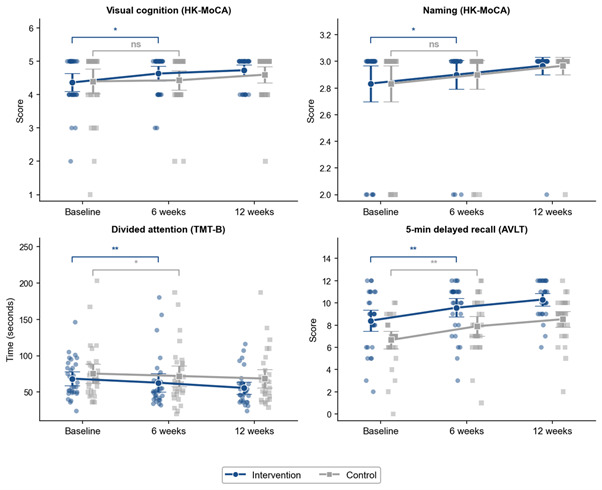
Trajectories of visual cognition, naming, divided attention, and 5-min delayed recall across the assessment time points. Significance of within-group change from baseline to 6 weeks is indicated above each group’s trajectory. AVLT: Auditory Verbal Learning Test; HK-MoCa: Hong Kong-Montreal Cognitive Assessment; TMT-B: Trial Making Test Part. *P<.05, **P<.01, and ***P<.001.

Changes in visual cognition, naming, divided attention, and 5-minute delayed recall at baseline, 6 weeks, and 12 weeks are shown in Figure 3 for the intervention group (blue circles) and the control group (gray circles). Each data point represents an individual participant’s score, with a horizontal offset added for clarity. Lines connect the group means at each time point, and error bars indicate 95% CIs. The intervention group showed significantly greater improvement than the control group in these functions.

**Figure 4.**
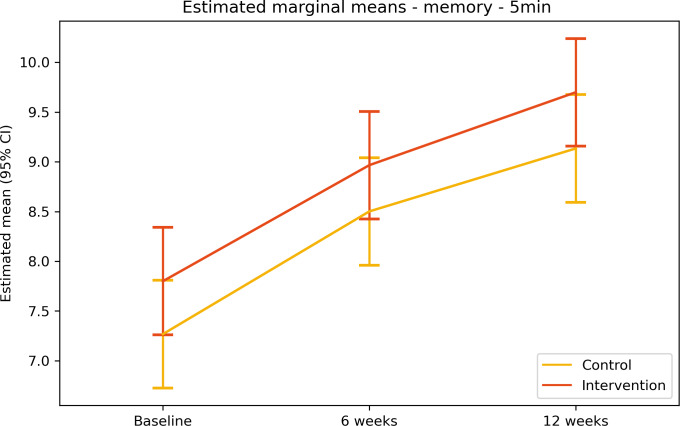
Estimated marginal means of 5-min delayed recall on the auditory verbal learning test (AVLT) for both intervention and control groups from baseline to 6 weeks and 12 weeks.

According to the linear mixed effects model analysis, a small to moderate group effect was observed for 5-minute delayed recall on the AVLT (Cohen *d*=0.38), with the fixed effects explaining 69% of the variance (marginal *R²*=0.69). Baseline scores strongly predicted follow-up performance (standardized β=0.78). A significant main effect of time was identified (*F*_2, 129.5_=25.70; *P*<.001), along with a significant group difference (*F*_1, 81.2_=3.997; *P=.*05), indicating that the intervention group showed better memory scores across visits. The time×group interaction was nonsignificant (*F*_2, 129.5_=0.022; *P=.*98; Figure 4).

Cognitive performance was similar between the groups at baseline. Post hoc pairwise comparisons showed that cognitive functions on HK-MoCA total scores in the intervention group increased significantly from baseline to week 6 (*P=.*03) and were maintained at week 12. In contrast, the control group showed a significant decline from week 6 to week 12 (*P=.*02) with no net change from baseline.

**Figure 5.**
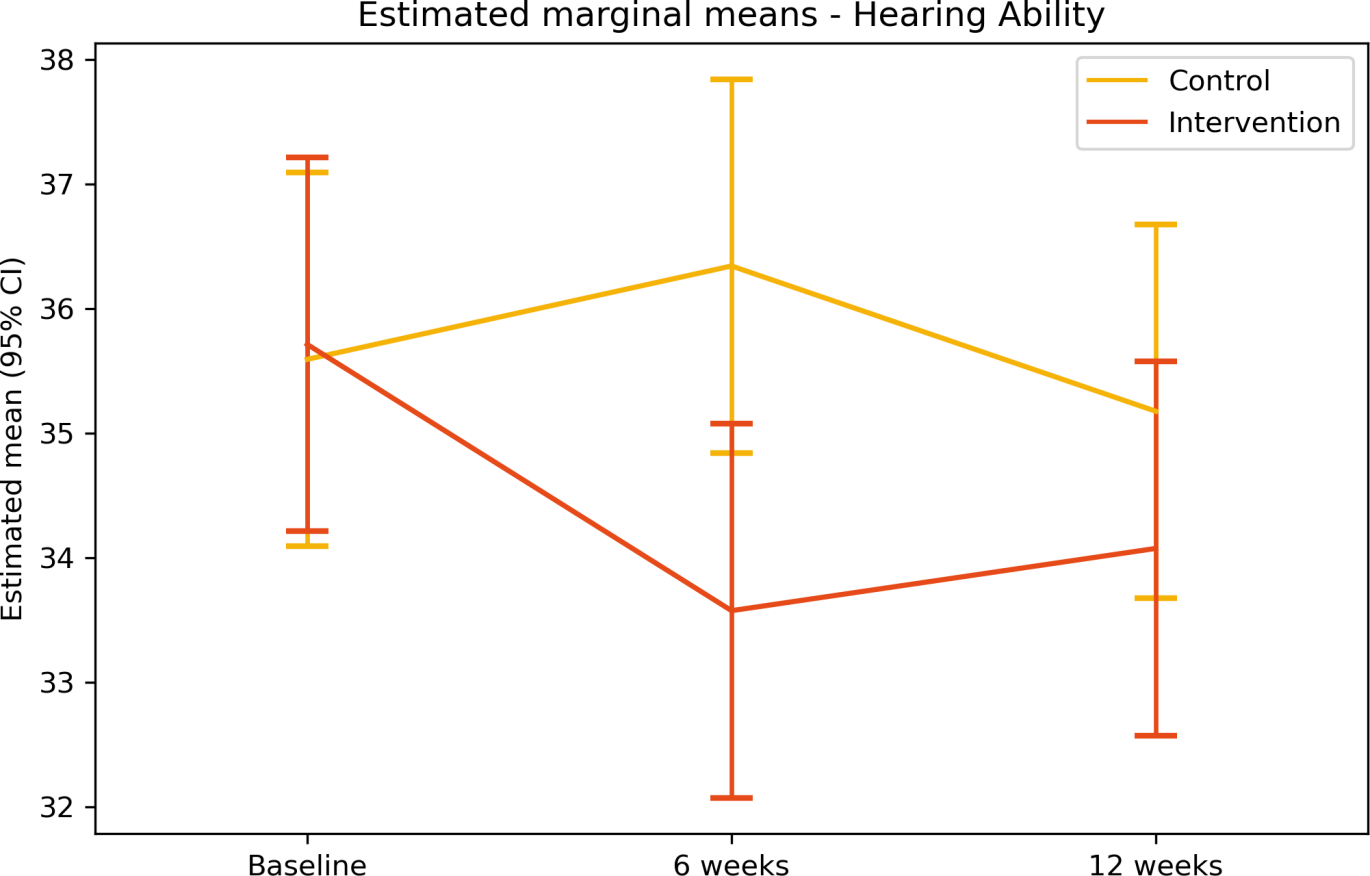
Estimated marginal means of hearing based on PTA for both intervention and control groups from baseline to 6 weeks and 12 weeks.

For hearing thresholds (Figure 5), the standardized baseline coefficient was strong (β=0.88), but the group effect remained minimal (Cohen *d*=0.17), with a marginal *R²* of 0.67. A significant time×group interaction was observed for left-ear thresholds (*F*_2, 117.0_=4.470; *P=.*01), indicating improved thresholds in the intervention group.

The Hearing Handicap Inventory for the Elderly total score showed a significant main effect of time (*F*_2, 112.6_=14.69; *P<.*001), with both groups reporting reduced perceived hearing handicap at weeks 6 and 12 compared with baseline. A significant group main effect was observed for the social subscale (*F*_1, 62.1_=4.81; *P=.*03), indicating that, on average, the intervention group reported lower perceived social hearing handicap than the control group, independent of time. There was no significant time×group interaction for the social subscale (*P=.*24).

### Qualitative Results

A total of 30 participants in the ACDT group were interviewed, and 3 key themes were generated based on the TFA [44]. No ethical issues were raised by participants. Opportunity costs refer to the trade-offs users may have to make when engaging with the intervention. None of them explicitly expressed such concerns. Instead, most rearranged daily activities to accommodate the training.

#### Theme 1: Intervention Coherence and Participants’ Affective Attitude Toward ACDT

Intervention coherence refers to the extent to which users understand the intervention, its purpose, and how it fits into their existing routines [44]. Participants generally valued the program’s alignment with their cognitive health goals. Daily routines involving auditory-cognitive training were embraced as brain-stimulating activities, with Participant 16 emphasizing, “I value daily hearing and cognition training. The system’s varied exercises provide consistent training.” Participant 38 similarly stated, “I train seriously to improve memory and hearing.” Some participants reported feeling nervous and under pressure when they first began the training, mainly because they were unfamiliar with the content and format. As Participant 35 mentioned, “At first, I was nervous because I had never done this before, but after a few sessions, I became more relaxed as I got used to the process.” Over time, most participants integrated ACDT into their daily routines, with Participant 5 reporting, “I do it every morning after I get up; it has become like homework,” and Participant 60 adding, “I attend the training every day even when traveling.”

Affective attitude focuses on how users feel about using the intervention [44]. Participants generally reported neutral to positive attitudes toward ACDT. Most described their training experience as “acceptable,” “quite interesting,” and “enjoyable” (Participants 18, 22, and 43). However, some participants felt that certain training components were insufficiently engaging. Participant 13 specifically requested more personalized content with interactive features, noting,

*For hearing training, I hope the content could be more interesting and personalized. I’m not interested in public transportation. The training should be more interactive, and I’d like immediate feedback.*

[Participant 13]

This preference for ecological relevance was further reinforced by reports that tasks involving familiar daily scenarios were easier and more interesting than those involving abstract content. As Participant 14 explained, “Daily activities like going to restaurants or supermarkets are more interesting and easier to remember.”

#### Theme 2: Perceived Benefits in Cognition, Information Acquisition, and Self-Awareness From ACDT

Most participants perceived that ACDT enhanced their abilities, particularly when they observed their own progress over time. Participant 60 described this as a source of motivation:

I think I do it happily as it is helpful to me. It turns out that when we see ourselves making progress, we can also challenge the difficulties.

[Participant 60]

Several participants reported improvements in attention and concentration during auditory tasks.

I notice the issue of concentration. When I want to learn something, I will know that I need to be more attentive and calm down to listen. This is the influence of the training on me.

[Participant 35]

Others described improvements in memory, mental clarity, and processing speed. Participant 5 observed, “I feel that my memory has improved because when doing math by heart, I often have to calculate the subtrahend and the addend.”

Participants also found ACDT informative when integrating real-life scenarios. Participant 4 stated, “Yes, in terms of hearing training, the content can help us. There is some information about healthy or daily life, like when you eat dim sum, or you should not eat fried foods,” while Participant 56 similarly described it as “very informative.*”*

Beyond functional improvements, participants demonstrated greater awareness of their hearing and attentional limitations, prompting adaptive changes in communication behaviors. As Participant 18 reflected,

*Sometimes it is not just about relying on hearing. I begin to realize that many times I cannot do certain things because I suddenly think of something else. So in daily life, I start to understand where my problems lie.* [Participant 18]

Sustainability concerns emerged, with Participant 56 expressing her concern about “maintaining gains after completing of this study.”

#### Theme 3: Perceiving ACDT as Less Burdensome With Enhanced Self-Efficacy

Most participants perceived the training as manageable, with several attributing this to prior life experience or visible self-improvement. As Participant 17 noted, “I don’t find it difficult as my previous job was very demanding,” while Participant 18 connected manageability to perceived benefits, “I feel no burden because it’s a training, where I can manage my own time to complete it.” Completing sessions often fostered a sense of accomplishment, as illustrated by Participant 34’s comment, *“*I feel confident after finishing tasks.” Participants also described progressive mastery of the auditory exercises, with several individuals (34, 42, and 57) noting “improved processing speed and accuracy.” Elements like competing voices were perceived as “both challenging and engaging” (Participants 5 and 34). While task difficulty occasionally prompted frustration, as Participant 55 acknowledged, “There is a sense of success when I answer correctly, but difficult tasks can be frustrating.” However, they maintained motivation through perceived utility. Participant 49 articulated this resilience, “The hardest parts are likely most useful; backward counting’s challenge makes it interesting.”

## Discussion

### Primary Cognitive Outcomes

This pilot RCT provides preliminary evidence for the feasible, acceptable, and potential efficacy of ACDT for older adults with ARHL. Participants in the ACDT group demonstrated significant improvements in focused attention, divided attention, naming, and visual cognition. However, the control group’s improvement in some cognitive measures suggests possible practice effects from repeated testing, highlighting the need for active control conditions in future trials. These findings extend evidence that combined auditory-cognitive training selectively enhances executive functions and visual processing, which are critical domains in dementia risk reduction [52]. This pattern converged with qualitative findings of “enhanced attention and concentration,” “improved memory and mental clarity,” and “increased processing speed” during daily activities. Mechanistically, results align with previous research that auditory training enhances attention in older adults with hearing loss [53], cognitive training improves domain-specific functions, and dual-task trainings strengthen executive control [34]. The gamified design likely amplified these effects through enhanced engagement and motivation, which is supported by participant feedback and studies showing gamification improves adherence and training outcomes in cognitive interventions [54]. Additionally, further research is warranted to examine the effects of ACDT on dual-task listening-cognitive abilities using an active control group, as poor dual-task performance is recognized as an early indicator of dementia [30].

Notably, the ACDT group exhibited improved 5-minute delayed recall with small-to-moderate group effects and maintained higher average scores across assessments than the control group. This finding is clinically significant given delayed recall is an established early marker for cognitive decline and dementia risk [55]. Furthermore, post hoc pairwise comparisons demonstrated that HK-MoCA total scores significantly increased from baseline to week 6 and were sustained at week 12 in the ACDT group, suggesting that ACDT may help stabilize global cognition over time.

### Auditory Threshold Findings and Possible Indicators of Training-Induced Neuroplasticity

Hearing threshold analysis revealed minimal overall between-group differences, which means that ACDT did not produce a significant improvement in PTA compared to the control group, and this finding is consistent with the recognized stability of peripheral hearing loss over short time intervals [56]. While the significant time×group interaction observed for left-ear thresholds may indicate training-induced neuroplasticity in central auditory pathways [57], these preliminary findings warrant confirmation in larger trials. Future studies should investigate whether specific training components can be optimized for bilateral benefits and determine if observed neuroplastic changes translate to measurable functional communication improvements.

### Perceived Hearing Handicap and Increased Awareness

Interestingly, the control group reported lower perceived overall hearing handicap but higher emotional hearing handicap than the ACDT group. One possible explanation is that the ACDT participants became more aware of their hearing challenges, leading to more accurate self-reporting. In contrast, the control group may have had lower insight into their hearing limitations, potentially resulting in underreporting. This interpretation aligns with qualitative findings and researcher observations during data collection, wherein ACDT participants frequently described increased awareness of hearing-related challenges in their daily lives. Previous research also supports that targeted interventions can improve auditory awareness, which may initially increase self-reported handicap but ultimately facilitate adaptive behavior change [58].

In our study, participants reported enhanced recognition of auditory and attentional limitations, which subsequently prompted adaptive modifications in communication behaviors. Many participants described integrating training into their daily routines and adopting compensatory strategies to improve attention and listening engagement, which may reflect a meaningful sign of behavior change. However, longitudinal investigation is required to verify sustainability. ACDT also fostered perceived accomplishment and strengthened self-efficacy, potentially encouraging sustained engagement in beneficial behavior changes. Collectively, these findings position ACDT as a catalyst for positive behavioral adaptations, benefiting not only participants but also their families.

In this study, no statistically significant changes were observed in social relationship or loneliness scores for either group. A possible explanation is that participants were comparatively young, most were married and living with a spouse or other family members, and they demonstrated a positive attitude toward adopting this new training to address their hearing problems. Future studies should test ACDT among individuals at higher risk of loneliness and social isolation.

### Enhancing Training Content and Interactivity Using Artificial Intelligence

While the intervention was generally well-received, participant feedback identified opportunities for enhancement through more personalized and interactive training content. Integration of artificial intelligence (AI) can dynamically tailor training modules to individual abilities and progression patterns, with real-time adjustments in difficulty and personalized feedback to optimize training efficacy while maintaining engagement. Machine learning algorithms could further enhance ecological validity by generating complex auditory environments that simulate real-world scenarios, improving training transferability to daily communication challenges. Incorporating AI-powered analytics could continuously monitor user performance trajectories, identify areas for improvement, and deliver targeted interventions to maximize auditory and cognitive gains. Future iterations of ACDT should prioritize the development of AI-enhanced features, including personalized content, interactive feedback, and real-time progress tracking. Engaging users and their families in the co-design process is essential to ensure that the platform meets their needs and preferences.

### Strengths and Limitations

This study demonstrates several strengths, including (1) multimodal assessment of auditory and cognitive outcomes, (2) ecologically valid gamified design that could enhance participant engagement, and (3) novel documentation of self-awareness as a facilitator for adaptive behavior change. However, several limitations warrant consideration: (1) the modest sample size and absence of longitudinal follow-up limit detection of subtle neuroplasticity effects, such as the observed hearing threshold trend; (2) the waitlist control design cannot exclude attention bias and isolate the specific efficacy of dual-task training; and (3) although ARHL has been reported to be more common, more severe, and to have an earlier onset in men than in women [59], the homogeneous participant characteristics regarding education level and hearing loss severity, along with a predominance of male participants, may restrict generalizability of our findings to broader ARHL populations.

### Conclusions

This pilot RCT suggests that ACDT is feasible and acceptable for older adults with ARHL, with preliminary evidence supporting its potential efficacy. The intervention appeared to benefit both auditory and cognitive domains, as well as self-awareness of hearing loss, which may facilitate adaptive behavioral adjustments. Future development could explore AI-enhanced personalization and interactivity to improve ecological relevance. Further trial with larger, more diverse samples, active control conditions, and longitudinal follow-up would help clarify ACDT’s sustained effects on auditory-cognitive health, dual-task listening-cognitive abilities, and real-world functioning.

## Supplementary material

10.2196/84083 Multimedia Appendix 1 Research protocol.

10.2196/84083 Multimedia Appendix 2 A log for checking study feasibility.

10.2196/84083 Checklist 1 CONSORT checklist.

## Abbreviations

ACDT: auditory-cognitive dual-task training
AI: artificial intelligence
ARHL: age-related hearing loss
AVLT: Auditory Verbal Learning Test
CONSORT: Consolidated Standards of Reporting Trials
HK-MoCA: Hong Kong-Montreal Cognitive Assessment
ICC: intraclass correlation coefficient
PTA: pure-tone average
RCT: randomized controlled trial
TFA: theoretical framework of acceptability
