# Supplementary material for: Web-Based Gamified Auditory-Cognitive Dual-Task Training for Older Adults With Age-Related Hearing Loss: Pilot Randomized Controlled Trial
Source: JMIR Aging. 2026 Jun 16;9:e84083. doi: 10.2196/84083 (PMC13271602; doi:10.2196/84083)
Supplement: Multimedia Appendix 1 [file aging-v9-e84083-s001.doc]

**
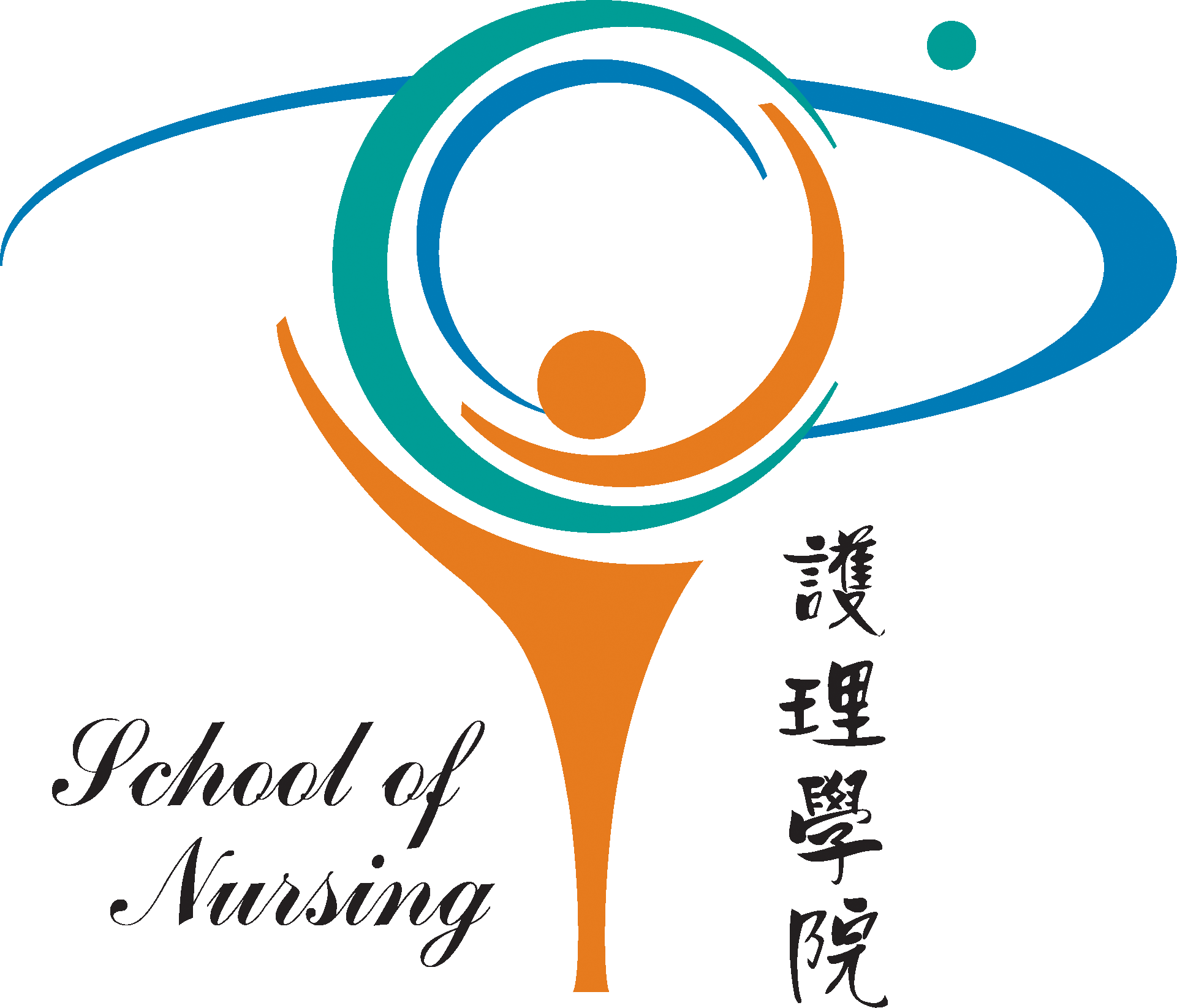

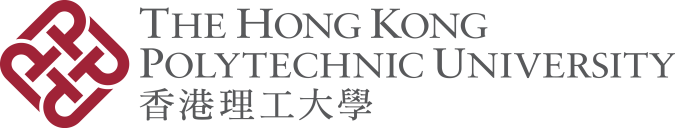
**

**Application for Start-up Fund**

**Auditory-Cognitive Dual-Task Intervention for Older Adults with Hearing Loss: A Pilot Randomized Controlled Trial**

1. Details of the Research Proposal
2. *Abstract of Research comprehensible to a non-specialist (a maximum of 400 words)*

**Background:** Age-related hearing loss (ARHL) is a common and irreversible condition that has been recently associated with cognitive decline and dementia. Hence, if ARHL is treated earlier, the risk of dementia might decrease. However, in China, only 0.8% of older adults with hearing loss wore hearing aids, and over two-thirds (67.5%) of older adults with ARHL in Hong Kong had either been formally diagnosed or treated. There is also limited information on the feasibility and efficacy of hearing loss interventions for older people in Hong Kong. It is important to develop hearing loss interventions that have the potential to improve cognitive functions among older people.

**Aims:** This study aims to assess the feasibility and acceptability of a new auditory-cognitive dual-task intervention (ACDT) for community-dwelling older adults with hearing loss, and to examine the preliminary efficacy of ACDT on their cognitive function.

**Study design and Methods:** This is mixed-model design, using a 2-arm, parallel-group, single-blinded, pilot randomized controlled trial (pilot RCT). A total of 60 community-dwelling older adults in Hong Kong who have mild to moderate hearing loss and normal cognitive performance will be recruited. Participants will be randomly assigned to the auditory-cognitive dual-task intervention group (ACDTG), and control group with no specific intervention (a “wait list” group) (CG). Each ACDTG participant will receive the intervention for 12 weeks (5 days x 60-min sessions per day). All participants in all groups will be assessed for cognitive function (primary outcome), social isolation, and loneliness, and hearing at baseline (T0), week-6 (T1) and Week-12 immediately after the intervention (T2). Post-intervention interviews will be conducted to obtain perspectives of participants in the ACDTG on the feasibility and acceptability of the ACDT intervention.

**Data analysis:** Participant characteristics and outcome variables will be analysed through descriptive statistics. Differences in cognition score and other outcomes across time points among the participant groups will be measured by Generalized Estimating Equations (GEE). The statistical software package IBM SPSS version 26.0 will be used. Content analysis will used to analyse the post-intervention interviews.

**Expected results:** ACDT will be feasible for implementation and acceptable for community-dwelling older adults with hearing loss. While ACDT will not be able to improve underlying hearing in ARHL, it will be more effective on improving participants’ cognitive function, social engagement and loneliness, and ability in information processing, interpretation and communication, than CG.

1. *Impact and objectives*

***(a maximum of 800 words for the long-term impact and project objectives)***

1. Originality and Long-term impact

This is the first pilot RCT study to investigate the feasibility and preliminary efficacy of an auditory-cognitive dual-task intervention (ACDT) for older adults with mild to moderate hearing loss. Since there was no dual-task training program, combing the auditory training with cognitive training documented for improving cognition in people with hearing impairment, further studies are needed to explore its feasibility, acceptability and preliminary efficacy. We hypothesize that dual-task training, through the integration of auditory and cognitive training would improve cognition and social engagement in older adults with hearing loss. Therefore, this study will be the first of its kind to assess an auditory-cognitive dual-task training intervention among people with ARHL.

The potential beneficiaries of the proposed research in the short term (1-3 years) will be older people with hearing loss in the community. The intervention aims to improve the cognitive functions, communication skills for people with ARHL and reduce risk factors for loneliness and social disengagement. In the medium term (4-10 years), with the ACDT applied in older people with ARHL, we anticipate that their communication skills, social relationships, cognitive functions will be supported and improved. Early management of ARHL and cognitive decline for older adults is more cost-effective than delayed treatment in terms of that older adults with untreated hearing loss experienced more inpatient stays, were at a greater risk of being readmitted to a hospital and had increased number of emergency department visits. The health system is anticipated to be benefited from deceased health service utilization by people with ARHL; thus, reducing healthcare costs. We will disseminate our findings through different platforms to policy makers, to the wider public in Hong Kong, and around the globe. Public policy makers are expected be aware and consider this problem and over the long-term period (over 10 years), the cognitive health of older adults or those with ARHL is anticipated to be improved, and such improvement might be sustained through self-performing ACDT by older adults at home to address hearing loss as a potential risk factor for social disengagement and cognitive decline. The findings of the pilot RCT of the intervention will support subsequent investigation in full scale RCT in the future. If older adults’ communication skills, social relationships and cognitive functions can be significantly improved, future investigations might examine the effectiveness of the intervention when applied to ageing populations with both hearing loss and cognitive decline. The knowledge of our study might also benefit other age groups with problem of hearing loss.

1. How this proposal will help your longer-term research plans

The findings of the pilot RCT of the ACDT will support subsequent investigation in a full scale RCT in the future.

1. Objectives

[Please list the objectives in point form]

The objectives of this study are:

- To assess the feasibility and acceptability of ACDT for community-dwelling older adults with ARHL;
- To investigate the preliminary efficacy of ACDT on the cognition of participants compared to those in the control group with no specific intervention (a “wait list” group) (CG), which will guide power calculation in a full-scale trial; and
- To investigate the preliminary efficacy of ACDT on social engagement, loneliness peripheral hearing (measured with pure tone audiometry) and hearing-related quality of life (measured by HHIE) of participants compared to control group.

1. *Background of research, research plan and methodology:*

[A maximum of seven A-4 pages in total for items (a) and (b).

Standard Format is: Times New Roman; 12 pt Font Size; 1-inch all round in Margin; and Single-line spacing]

1. Background of research

The issue of age-related hearing loss (ARHL)

Presbycusis, or age-related hearing loss (ARHL) is a gradual, progressive and bilateral symmetric sensorineural hearing loss that is associated with aging (Chern & Golub, 2019). The recent Global Burden of Disease (GBD) states that hearing loss is the third leading cause of years lived with disability, which is a major concern for global healthy aging (Collaborators GDaIIaP, 2017). According to the World Report on Hearing, approximately 70% of people over the age of 70 (>8.3 m people) have ARHL (World Health Organisation, 2021). Limited epidemiological data regarding the prevalence of ARHL is available in the Chinese population. In the 2013 census report of Hong Kong on disabilities and chronic diseases, the number of people with hearing difficulty increased from 92,200 (or 1.3% of total population) in 2007 to 155,200 (or 2.2% of total population) in 2013. There were 128,900 people aged 60 and above (21.8% of total population) reported hearing difficulty and the prevalence of ARHL was higher among people over the age of 70 and above (14.2% of total population) (Census and Statistic Department, 2013). This population-based data is, however, believed to have seriously underestimated the prevalence of hearing loss in Hong Kong, due to issues with sampling and data collection with the absence of a reliable central register of disabilities. Our project team recently accomplished a cross-sectional study in 11 community centers in Hong Kong assessing the intrinsic capacity of older adults through the WHO Integrated Care for Older People (ICOPE) assessment framework. Our results documented that 27.9% of participants (82/304) aged 60 and above have hearing impairment and 25.5% (75/304) have cognitive decline (Leung et al., 2022).

ARHL is irreversible and hearing interventions mainly include hearing aids and cochlear implantation. Despite the high prevalence of ARHL, treatment remains vastly underutilized globally (Goman & Lin, 2018). In China, only 0.8% of older adults with hearing loss wore hearing aids (Heine, Browning, & Gong, 2019). In Hong Kong, over two-thirds (67.5%) of older adults with ARHL had either been formally diagnosed or treated (Census and Statistic Department, 2013). The current hearing healthcare service for older people in Hong Kong is lack of directed initiatives and information on hearing aid adoption among people with ARHL is absent (Ng & Loke, 2015). Older people with financial difficulties may apply for the hearing aid subsidies from the government but have to encounter a long waiting time (months or even years) and short average consultation time per appointment (Ng & Loke, 2019).

Previous research about ARHL and cognitive impairment

Numerous studies, including systematic reviews and meta-analyses, have shown the associations of ARHL with cognitive decline and increased risk of dementia (At et al., 2015; Loughrey et al., 2018; Uchida et al., 2016; Yeo et al.' 2022). Cohort studies have also demonstrated that even a mild level of ARHL could increase the long-term risk of cognitive decline and dementia in cognitively-intact individuals who reported hearing loss at baseline (Amieva et al., 2015; Deal et al., 2016; Deal et al., 2015; Fritze et al., 2016). Cross-sectionally, lower scores on measures of mental status, memory, and executive function were more likely to be reported by people with more severe ARHL (Lin et al., 2011; Saji et al., 2021). The hazard ratio for incident dementia increased to 4.9 in older adults with greater hearing loss compared to those without a hearing problem (Lin et al., 2014). Greater ARHL is also associated with increased social isolation (Mick et al., 2014) and loneliness (Applebaum et al., 2019) among older adults. According to a recent systematic review, ARHL is associated with decreased communication, fewer social interactions, and disengagement in older adults (Shukla et al., 2020b). Noise from the external environment at home, such as music, television, radio, announcement systems, and surrounding conversations could diminish the ability of older adults to hear others and engage in conversations (Ludlow et al., 2018). Older people with hearing loss are more likely to be left out of conversations or to avoid social networking (Aberdeen & Fereiro, 2014). Communication difficulties for older people with hearing loss can be intensified when the person also has cognitive impairment (Ludlow et al., 2018). As Rutherford et al. (2018) have suggested, well-designed studies are imperative to investigate if hearing loss interventions could serve as means to improve cognitive outcomes in older adults.

The importance of early management of age-related hearing loss and cognitive decline

Dementia is a primary global health concern affecting approximately 50 million people, with the number anticipated to increase to 152 million in 2050 (Prince et al., 2015). The Lancet International Commission on Dementia, Prevention, Intervention, and Care estimated that mid-life hearing loss, if treated, might decrease the risk of dementia by nine percent (Livingston et al., 2020). Cognitive decline is a stage preceding dementia that can be improved or delayed by applying appropriate interventions. Early intervention for cognitive decline is crucial and more likely to substantially delay the onset of dementia (Ryu, 2018). Moreover, early management of ARHL and cognitive decline for older adults is more cost-effective than delayed treatment (Shah et al., 2016). In the United States, a 10-year cohort study conducted by our project team suggested that untreated ARHL was associated with higher total healthcare costs of US$22,434 or 46% higher compared with costs for residents without hearing loss (Reed et al., 2019). Older people with untreated hearing loss experienced more inpatient stays and were at a greater risk of being readmitted to a hospital after 30 days at the 2-, 5-, and 10-year timepoints (Reed et al., 2019). In China, older adults with fair or poor hearing reported spending more time in hospitals than older people who reported excellent or very good hearing (Ye et al., 2021). In the UK, ARHL was found to predict domains f cognition, and subsequently to have more service use in the 1-year follow-up (Crealey & O'Neill, 2020).

Existing hypotheses of etiological mechanisms between ARHL and cognitive decline

The underlining causal mechanisms underpinning the connection between ARHL and cognitive decline are not well understood (Uchida et al., 2019). The commonly reported hypotheses of etiological mechanisms between ARHL and cognitive decline (see Figure 1) based on previous basic science studies include: 1) the cognitive load hypothesis (impaired hearing may contribute causally to cognitive decline through either the degradation of auditory signals or the depletion of information for perceptual processing) (Wingfield et al., 2005); 2) the overdiagnosis or harbinger hypothesis (*decreased hearing capability impacts performance on some specific neuropsychological tests, rather than cognitive function*) (Dupuis et al., 2015); 3) the cascade hypothesis (*hearing loss could lead to social disengagement, social isolation, or depressive symptoms, which accelerates the rate of brain atrophy*) (Gopinath et al., 2009; F.R. Lin et al., 2014); and 4) the common cause hypothesis (*it assumes that there is a common factor, which is associated with age-related deterioration in cognitive and hearing loss in the aging brain*) (Stahl, 2017; Wayne & Johnsrude, 2015). These possible mechanisms are not mutually exclusive and future well-designed studies are needed to support the actual underlying mechanisms.

Cognitive training and auditory training for promoting cognition

The Scaffolding Theory of Ageing and Cognition-revised (STAC-r) is a conceptual model of cognitive aging that integrated evidence from structural and functional neuroimaging to explain how the combined effects of adverse and compensatory neural processes and life-course factors produce varying levels of cognitive function (Park & Reuter-Lorenz, 2014) (see Figure 2). The model suggests that the brain builds protective “scaffolds” in response to the age-related neural changes such as brain shrinkage, decreased white matter integrity, and decreased dopamine receptors. Social/intellectual engagement, cognitive training, new learning, and exercise are associated with ‘scaffolding enhancement’ and lead to beneficial effects on cognitive function.

Cognitive training usually involves guided and repeated practice on a set of tasks designed to solicit targeted cognitive functions (Ball et al., 2002). Cognitive training is evidenced to improve older adults’ overall cognition, executive function, memory, attention, and visuospatial ability (Chiu et al., 2017; Mewborn et al., 2017). Van Balkon et al. (2020) also suggested that cognitive training was able to functionally stimulate several brain regions, such as the hippocampus and frontoparietal networks. Cognitive training has been recommended in healthy older adults to improve cognitive functioning (Phirom et al., 2020; Yu et al., 2021) and has been suggested as an effective strategy to prevent cognitive decline in older people with mild cognitive impairment (MCI) (Campbell et al., 2022; Lipardo & Tsang, 2018).

Auditory training interventions were previously designed for new hearing aid users to interpret newly-amplified speech (Burk et al., 2006). Computer-based auditory training programs focus more on using auditory stimuli to improve perceptual and cognitive abilities that translate into better speech-communication abilities for individuals with hearing loss (Henderson Sabes & Sweetow, 2007). It was suggested that auditory training program for people with ARHL may recruit additional (frontal) cognitive resources to compensate for diminished auditory perception and to support their attention and working memory capacity (Goh & Park, 2009). Moreover, a recent review underlined that hearing loss could prohibit meaningful interaction and engagement with others (Shukla et al., 2020), and that auditory training might be able to enhance communication and engagement (Mamo & Wheeler, 2021) and improve the ‘scaffolding’ (Park & Reuter-Lorenz, 2014). Though preliminary evidences have demonstrated the beneficial effects of managing ARHL on cognitive function, randomized clinical trials are needed to examine whether combing auditory and cognitive training could help to reduce the risk of cognitive impairment (Rutherford et al., 2018).

Dual-task training (simultaneous auditory and cognitive training)

Dual-task training involves the simultaneous performance of two tasks that can be assessed separately and have distinct purposes (McIsaac et al., 2015). Dual-task interventions incorporating various cognitive exercises is highly promising and could maximize the potential benefits of lifestyle activities on cognition (Parial et al., 2022). Moreover, poor dual-task performance is suggested as an early indicator of dementia and can greatly impact activities of daily life (Schwenk et al., 2010). Individuals who perform poorly on dual-task tests (e.g., hearing while engaged in cognitively demanding tasks) are also likely to have interpretation and communication problems. Finding ways to improve dual-task ability may reduce functional decline and mediate effective commination for people with ARHL (Ludlow et al., 2018). Since there was no dual-task training program, combing the auditory training with cognitive training documented for improving cognition in people with hearing impairment, further studies are needed to explore its feasibility, acceptability and preliminary efficacy (Ferguson & Henshaw, 2015; Lawrence et al., 2018b). We hypothesize that dual-task training, through the integration of auditory and cognitive training would improve cognition and social engagement in older adults with hearing loss. Therefore, this study will be the first of its kind to assess an auditory-cognitive dual-task training intervention among people with ARHL.

1. Research plan and methodology

**Aims and hypotheses of the study**

This study aims to assess the feasibility and acceptability of a new auditory-cognitive dual-task intervention (ACDT) for community-dwelling older adults with hearing loss, and examine the preliminary efficacy of ACDT on improving their cognitive functions. The objectives of this study are:

- To assess the feasibility and acceptability of ACDT for community-dwelling older adults with ARHL;
- To investigate the preliminary efficacy of ACDT on the cognitive functions of participants compared to those in the control group (a “wait list” group) (CG), which will guide power calculation in a full-scale trial; and
- To investigate the preliminary efficacy of ACDT on social engagement, loneliness peripheral hearing (measured with pure tone audiometry) and hearing-related quality of life (measured by HHIE) of participants compared to control group.

The hypotheses of this study are:

- ACDT is feasible for implementation and acceptable for older adults with hearing loss living in the community;
- ACDT is more effective on improving the participants’ cognitive functions compared to those in CG;
- While ACDT will not be able to improve underlying hearing in ARHL, it will be more effective on improving participants’ social engagement and loneliness, and ability in information processing, interpretation and communication, than CG.

**Participants (with justification on the sample size)**

In this study, a total of 60 community-dwelling Chinese older adults in Hong Kong will be recruited.

*The inclusion criteria are:*

- aged 60 and above living in the community;
- have mild to moderate hearing loss with a pure-tone average (PTA) between 25 and 60 dB in both ears (average hearing threshold at 0.5, 1, 2, and 4 kHz (Informal Working Group on Prevention of Deafness and Hearing Impairment Programme Planning & World Health Organization, 1991) measuring by audiometer with headphones in a quite listening environment, no hearing aid use within the past 6 months;
- with normal cognitive performance (MoCA score ≥26); and
- are willing to and capable of providing informed consent and complying with study procedures.

*Exclusion criteria include:*

- have a history of psychosis, mania, bipolar disorder, substance use disorder or have current suicidal ideation;
- with severe or unstable medical illness, significant retrocochlear pathology or organic lesion responsible for hearing loss;
- a diagnosis of probable Alzheimer’s disease, vascular dementia, FTD, or Parkinson’s Disease; and
- taking medications such as antidepressants, sedatives, or antiepileptics that may affect cognition.

Sample size calculation As a pilot RCT study, a minimum sample size between 24 and 50 have been recommended (Hooper, 2014; Julious, 2005). Assuming an attrition rate of 20%, we decide a total sample size of 60 (20 per arm) for this study by using the *G* power software. According to the recent meta-analysis, the effect size of using auditory training for adults with hearing loss on cognitive functions was *d*=0.34 (Lawrence et al., 2018). To plan for a main trial designed with 80% power and two-sided 5% significance, a sample size of 60 is recommended to be able to find an effect size of 0.3. All participants in the ACDT group will be invited to participate in individual interviews after the intervention.

**Methods**

This is a 2-arm, parallel-group, single-blinded, pilot randomized controlled trial (pilot RCT).

**Study design**

Participants will be randomly assigned to the auditory-cognitive dual-task intervention group (ACDTG), and control group with no treatment (CG) by an independent statistician. Each ACDTG participant will receive 12 weeks intervention (5 days x 60-min sessions per day). According to previous auditory training and dual-task cognitive training intervention studies on improving cognition of older adults (Lawrence et al., 2018a; Parial et al., 2021), an intervention duration of 12 weeks is needed. Primary (global cognition) and secondary outcomes (hearing, social isolation, and loneliness) will be assessed at baseline and after the 12-week intervention.

A pilot study also aims to identify crucial parameters for the whole procedure of the main trial, especially when introducing new interventions. For obtaining a comprehensive picture, we will utilize both quantitative and qualitative approaches in data collection. Quantitative method will provide information about the recruitment and retention rates, implementation fidelity, adherence, and acceptability. Qualitative individual interviews with participants in the ACDT group will be conducted immediately after the intervention by trained research assistants according to the semi-structured interview guide. Some open-ended and probing questions will be asked following a semi-structured interview guide (**Table 1**):

1. Procedures

Participants will be recruited through 2-3 elderly community centres of the Yan Chai hospital. Flyers (in Chinese) will be distributed in places where Chinese late‐life immigrants frequented. In addition, we will arrange 2-3 information sessions to answer people's questions related to the research project. Individuals who agreed to participate and signed the consent form will be invited to complete the hearing assessment for eligibility screening and baseline scale measurements prior to randomization. Participants allocated to the intervention groups will have a briefing session about the training program. At Week-6 and Week-12, participants of two groups will complete the same measures administered at baseline. Individual interviews will also be conducted among the participants in the ACDT group.

1. Intervention protocol

The study team comprises of experts in age-related hearing loss, auditory training, cognitive health of older people, and dual-task cognitive training. We developed the intervention with each member’s expert input and the evidence from literature review. **Table 2** shows the intervention protocol.

***Auditory-cognitive dual-task training (ACDT)***

The new auditory-cognitive dual-task training (ACDT) is a 12-week program (5 times/week, 60 minutes per day). Auditory training (i.e., speech-in-noise training, rapid speech training and competing speaking training) will be incorporated with cognitive exercises involving the domains of executive function, perceptual-motor ability, memory, and complex attention. The components of auditory training are designed based on an auditory training and aural rehabilitation program LACE^TM^ (Listening & Communication Enhancement) (Sweetow & Henderson-Sabes, 2004) developed by our study team member. The cognitive training was developed and tested feasible and valid in our study team’s previous dual-task Zumba cognitive training (Parial et al., 2022). **Table 2** presents details of the intervention protocol.

The instructions of training session will be digitally recorded in Cantonese and Putonghua before the intervention. The training session will be started with a 5-minute volume adjustment to achieve a comfortable listening level for participants, incorporating attention/orientation training, such as asking questions to participants about orientation to person (identifying their names), time (date, month, or year), and place (current location). The auditory training will be last for 25 mins. One of the most common and frustrating symptoms of hearing loss is difficulty hearing a speaker in a noisy environment. The ***Speech-in-Noise exercises***, designed in three steps and facilitated by a trained instructor, will train participants’ brain and strengthen his/her brain’s auditory processing, helping he/she better filter out background noise to focus on the voice or voices he/she is listening to (Sweetow & Henderson-Sabes, 2006). For older people with hearing loss, difficulty understanding rapid speech is one of the most common hearing complaints. ***The Rapid Speech training*** is also designed in three steps for training the brain to focus on fast talkers and support older adult to understand more of what he/she hear (Sweetow & Henderson-Sabes, 2006). In addition, for people with ARHL, listening when there are multiple speakers talking can be difficult. ***The Competing Speaker training*** can train the brain to ignore a competing speaker’s voice while focusing on the conversation he/she is engaged in through two steps (Sweetow & Henderson-Sabes, 2006). The cognitive training will be last for 25 minutes, incorporating cognitive tasks of executive function (forward and backward serial counting); perceptual-motor ability (doing arm-clock positions based on prompts); memory (forward and backward recall of word/number series); and complex attention (forward and backward spelling). Each participant will perform each task individually. A 5-min break, the instructor will provide some paper cards with communication tips and strategies ranging from managing the acoustical environment to assertive listening skills to participants, they will read those cards and also complete a memory training to end each session.

***Control group*** will be provided with no specific treatment. This is a “wait list” group, participants can choose either the Auditory-cognitive dual-task training or the cognitive training after 12 weeks.

1. Outcome measures

**A**ll participants (in the ACDTG and CG groups) will be assessed at baseline (T0), Week 6 (T1), and Week 12 (immediately after the intervention) (T2) by a trained research assistant who is blinded to the group allocation.

***Primary outcome***: *Global cognition* will be measured by a comprehensive neuropsychological test battery. MoCA measures multiple cognitive domains, including attention, orientation, concentration, language, memory, executive functions and visuo-spatial skills. The MoCA was translated and validated in Hong Kong among older Chinese people with good sensitivity and specificity (Yeung et al., 2014). HK-MoCA score≥23 is considered as normal cognition, score 20, 21, 22 refers to mild cognitive impairment, score ≤19 is dementia. The Chinese Auditory Verbal Learning Test, the Chinese Trail Making Test Part A, the Chinese Trail Making Test Part, the category verbal fluency tests (animal, fruit, and vegetable), and the grooved pegboard for both dominant hand and nondominant hand. These tests were validated in the Chinese population (Lam et al., 2011).

***Secondary outcomes***

1. *Hearing:* will be measured by an audiometer with headphones and sound level meter (to measure ambient noise). Chinese version of the Hearing Handicap Inventory for the Elderly (HHIE; Ventry and Weinstein, 1982) and the five-point Communication Scale for Older Adults (CSOA; Kaplan et al, 1997) will be utilized.
2. *Social isolation:* will be measured by validated abbreviated 6-item Chinese version of the Lubben Social Network Scale (LSNS), which was developed specifically for use among older adults and shown to be both reliable and valid (Chang et al., 2018).
3. *Loneliness:* will be measured by the 6-item De Jong Gierveld loneliness scale (Chinese version) (Leung et al., 2008).
4. Feasibility outcomes

***Intervention acceptability***: Participants’ experience of participating in the ACDT intervention and their perceptions/acceptability of the intervention will be collected. Each interview will take approximately 30 minutes, and the session will be recorded digitally. Data collection will cease when data saturation is reached.

***Recruitment*:** Time taken for recruitment; percentage of eligible subjects agreeing to participate

***Retention*:** Number of participants completing the study versus the total number of people who started; reasons for withdrawal from the study

***Adherence*:** number of sessions that participants attended divided by the total number of sessions

1. Assessment of Intervention fidelity

Intervention fidelity will be monitored on a bi-weekly basis by a trained research assistant to make sure interventions are following protocol. An instructor trained by the study team will implement the ACDT sessions at community centres. This includes two steps: First, before starting the study interventions, our study team will conduct an orientation and practice training to enable the participants to be familiar with the process. On-site checking if the interventions are conducted to ensure that it is operating properly. Second, Two trained volunteers will perform safety monitoring and collect participants’ feedback in each session. A check list for the on-site checks will be used to document the fidelity process.

**Ethical approval**

Ethical approval will be obtained from the Human Subjects Ethics Sub-committee of the Hong Kong Polytechnic University. Participation in the study is voluntary, and people who declined will not be disadvantaged for community services. Trained research personnel will explain study details to potential participants to obtain informed consent before screening and participation. Only study team members have the access to participants’ information. The control group will be allowed to choose cognitive training or dual-task training after the conclusion of the study.

**Data processing and analysis**

Quantitative data: Characteristics and outcome variables of the participants will be reported in the descriptive analysis. Differences in cognition score, hearing, social isolation and loneliness outcomes across time points among ACDTG and CG will be assessed by the Generalized Estimating Equations (GEE). GEE could provide more efficient analysis for studies with small sample size, such as this pilot RCT and is more robust to missing data than traditional ordinary least squares (OLS) estimation models. As it relaxes the distribution assumption, GEE could also facilitate an inclusive analysis of study outcome variables that are not normally distributed. Effect sizes, which could be used for power calculation in the main trial, will be reported using Cohen’s *d* (≥0.2 = small; ≥0.5 = moderate; ≥0.8 = large). The statistical software package IBM SPSS version 26.0 will be used.

Qualitative data from individual interviews**:** The recorded interviews will be transcribed verbatim. Content analysis will then be used to analyse the transcribed interviews in relation to the feasibility and acceptability of the ACDT intervention. Two research team members will perform the analysis independently and their findings will be compared/contrasted. Differences will be resolved through group discussion to determine the final set of qualitative themes.

1. A maximum of two non-text pages of attached diagrams, photos, charts, and table etc, if any.


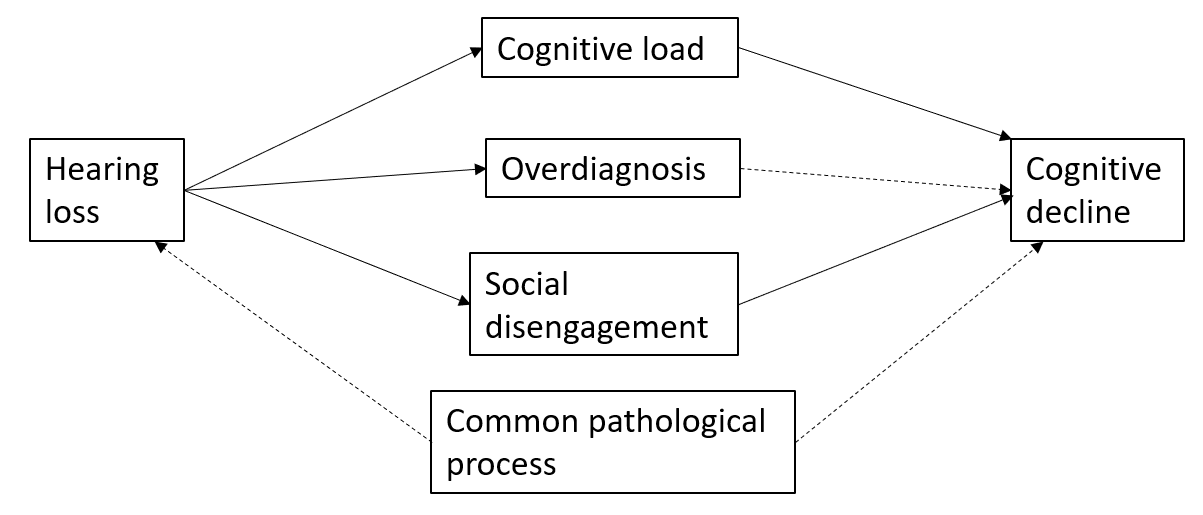


**Figure 1**. The existing hypotheses on etiological mechanisms between ARHL and cognitive decline. (Solid lines indicate potential mechanistic pathways. Dashed lines indicate potential underlying common factors.)


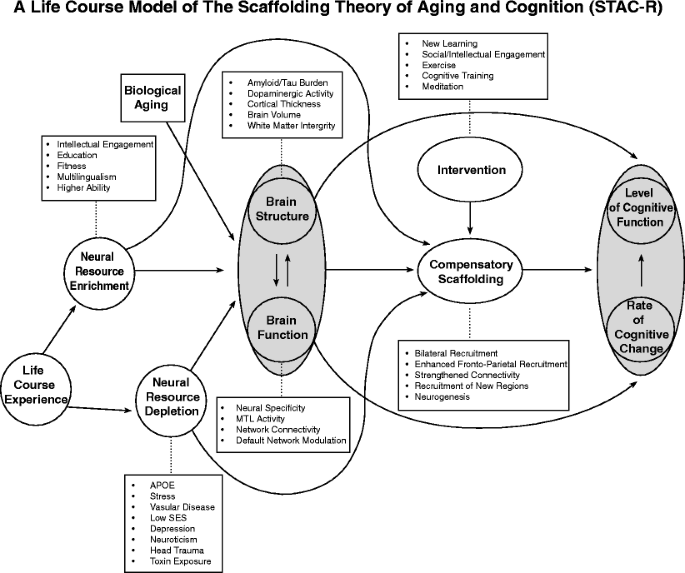


**Figure 2.** A conceptual model of the Scaffolding Theory of aging and cognition-revised (STAC-r) (Park & Reuter-Lorenz, 2014)

**Table 1.** Interview semi-structured guide

| **Example of questions** |
| --- |
| - Please tell me about your general experience of attending ACDT sessions? - Have you noted any changes in your health/daily life after participating in ACDT? Do you feel these are because of the training? - Which ADCT task do you think is the most helpful and which one is not the most helpful? Can you tell me why you selected these tasks? What made them useful or not useful? - Please tell me about your experience with the auditory training in this program? - Please tell me about your experience with the cognitive training in this program? - Please tell me about your experience performing the auditory and cognitive trainings simultaneously? - What difficulties or problems did you experience when you are using it? - Would you like to keep doing this training in the future at your home? - What the instructors or facilitators did that were most/least helpful? - How do you think about the group setup of this ADCT program? - Do you have suggestion for us to improve the ADCT program? |

**Table 2.** Intervention protocol

| **Auditory component** | **Cognitive component** |
| --- | --- |
| Warm-up session (5 minutes) | |
| Each participant will have earphones attached to a digital device. The volume of digital recorded instruction will be adjusted to achieve a comfortable listening level for participants | ***Orientation training:***   - 1) Each participant will be asked to state his/her full name - 2) Each participant will be asked to state about current date, month, year, and location |
| Training session (50 minutes) | |
| Approximately 2000 sentences (in Chinese) in different subject topics (e.g., money matters, exercise, or health) will be used as stimuli.  ***Speech-in-Noise exercise:*** 1) The subject will receive an audio-only presentation of the first sentence in a noisy environment and asked to repeat; 2) Receive both audio and visual representation of the sentence, and provide immediate feedback; 3) Receive the audio presentation only and think about the sentence as the auditory stimuli are repeated, paying close attention to the sounds that were not initially heard.  ***Rapid Speech training:*** 1) Hear a sentence and repeat; 2) See the sentence in writing and indicate whether understand every word of the sentence. 3) Increase the speed of the sentences and practice.  ***Competing Speaker training:*** 1) Listen to one of three voices as target voice: a male, female, or child; 2) The other voice will be the competing voice and will be presented louder or softer to make as a challenge. | ***Executive function:***1) Serial counting forward and backward as a group based on the proposed intervals every 4 weeks (1,2…); 2) Counting forward and backward from one participant to another  ***Perceptual-motor/visuospatial ability:*** Demonstrating basic clock positions using both arms when the instructor mentioned a particular time  ***Memory:*** Repetition of number/word series (forward and backward) with progressive length every 4 weeks (3-, 4-, 5-number/word series);  ***Attention:*** Spelling words (forward and backward) within the same category (local animals) with progressive length every 4 weeks (3-, 4-, 5-letter words); |
| Wrap-up session (5 minutes) | |
| ***Effective Communication Strategies:*** Receive communication tips and strategies ranging from managing the acoustical environment to assertive listening skills. | ***Memory:*** 1) Recall of arm clock positions performed in the visuospatial task; 2) Recall of numbers and words mentioned during the memory task |

1. Reference (a maximum of three pages for references is allowed for listing the publications cited. All references should be provided in full and include all authors.)

Aberdeen, L., & Fereiro, D. (2014). Communicating with assistive listening devices and age-related hearing loss: perceptions of older Australians. Contemp Nurse, 47(1-2), 119-131. https://doi.org/10.5172/conu.2013.4273

Amieva, H., Ouvrard, C., Giulioli, C., Meillon, C., Rullier, L., & Dartigues, J. F. (2015). Self-reported hearing loss, hearing aids, and cognitive decline in elderly adults: a 25-Year Study. J Am Geriatr Soc, 63, 2099–2104.

Applebaum, J., Hoyer, M., Betz, J., Lin, F. R., & Goman, A. M. (2019). Long-term subjective loneliness in adults after hearing loss treatment. International journal of audiology, 58(8), 464–467. https://doi.org/10.1080/14992027.2019.1593523

AT, J., Dias, A., & Philp, I. (2015). Identifying common impairments in frail and dependent older people: validation of the COPE assessment for non-specialised health workers in low resource primary health care settings. BMC Geriatr, 15, (123). https://doi.org/10.1186/s12877-015-0121-1

Ball, K., Berch, D. B., Helmers, K. F., Jobe, J. B., Leveck, M. D., Marsiske, M., Morris, J. N., Rebok, G. W., Smith, D. M., Tennstedt, S. L., Unverzagt, F. W., Willis, S. L., & Advanced Cognitive Training for Independent and Vital Elderly Study Group. (2002). Effects of cognitive training interventions with older adults: a randomized controlled trial. JAMA psychiatry, 288(18), 2271–2281. https://doi.org/10.1001/jama.288.18.2271

Burk, M. H., Humes, L. E., & Amos, N. E. (2006). Effect of training on word-recognition performance in noise for young normal-hearing and older hearing-impaired listeners. Ear Hear, 27, 263–278.

Campbell, E. B., Delgadillo, M., Lazzeroni, L. C., Louras, P. N., Myers, J., Yesavage, J., & Fairchild, J. K. (2022). Cognitive Improvement Following Physical Exercise and Cognitive Training Intervention for Older Adults with MCI. The journals of gerontology. Series A, Biological sciences and medical sciences, glac189. https://doi.org/10.1093/gerona/glac189

Census and Statistic Department. (2013). Special Topics Report No. 62.

Chang, Q., Sha, F., Chan, C. H., & Yip, P. (2018). Validation of an abbreviated version of the Lubben Social Network Scale ("LSNS-6") and its associations with suicidality among older adults in China. PlOS ONE, 13(8), e0201612. https://doi.org/10.1371/journal.pone.0201612

Chern, A.,

Golub, J. S. (2019). Age-related Hearing Loss and Dementia. Alzheimer disease and associated disorders, 33(3), 285–290. https://doi.org/10.1097/WAD.0000000000000325

Chern, A., & Golub, J. S. (2019). Age-related Hearing Loss and Dementia. Alzheimer disease and associated disorders, 33(3), 285–290. https://doi.org/10.1097/WAD.0000000000000325

Chiu, H. L., Chu, H., Tsai, J. C., Liu, D., Chen, Y. R., Yang, H. L., & Chou, K. R. (2017). The effect of cognitive-based training for the healthy older people: A meta-analysis of randomized controlled trials. PlOS ONE, 12(5), e0176742. https://doi.org/10.1371/journal.pone.0176742

Collaborators GDaIIaP. (2017). Global, regional, and national incidence, prevalence, and years lived with disability for 328 diseases and injuries for 195 countries, 1990–2016: a systematic analysis for the Global Burden of Disease Study 2016. Lancet, 390(10100), 1211-1259. https://doi.org/10.1016/S0140-6736(17)32154-2

Deal, J. A., Betz, J., Yaffe, K., & The Health ABC Study Group. (2016). Hearing impairment and incident dementia and cognitive decline in older adults: the Health ABC Study. J Gerontol A Biol Sci Med Sci https://doi.org/10.1093/gerona/glw069.

Deal, J. A., Sharrett, A. R., & Albert, M. S. (2015). Hearing impairment and cognitive decline: a pilot study conducted within the atherosclerosis risk in communities neurocognitive study. Am J Epidemiol 181, 680–690.

Dupuis, K., Pichora-Fuller, M. K., Chasteen, A. L., Marchuk, V., Singh, G., & Smith, S. L. (2015). Effects of hearing and vision impairments on the Montreal Cognitive Assessment. Neuropsychology, development, and cognition. Section B, Aging, neuropsychology and cognition, 22(4), 413–437. https://doi.org/10.1080/13825585.2014.968084

Ferguson, M., & Henshaw, H. (2015). Auditory training can improve working memory, attention, and communication in adverse conditions for adults with hearing loss. Frontiers in Psychology, 6, 1–7. https://doi.org/10.3389/ fpsyg.2015.00556

Fritze, T., Teipel, S., Óvári, A., Kilimann, I., Witt, G., & Doblhammer, G. (2016). Hearing impairment affects dementia incidence. An analysis based on longitudinal health claims data in Germany. PlOS ONE, 11(e0156876).

Goh, J. O., & Park, D. C. (2009). Neuroplasticity and cognitive aging: the scaffolding theory of aging and cognition. Restorative neurology and neuroscience, 27(5), 391–403. https://doi.org/10.3233/RNN-2009-0493

Goman, A. M., & Lin, F. R. (2018). Hearing loss in older adults - From epidemiological insights to national initiatives. Hearing research, 369, 29–32. https://doi.org/10.1016/j.heares.2018.03.031

Gopinath, B., Wang, J. J., Schneider, J., Burlutsky, G., Snowdon, J., McMahon, C. M., Leeder, S. R., & Mitchell, P. (2009). Depressive symptoms in older adults with hearing impairments: the Blue Mountains Study. Journal of the American Geriatrics Society, 57(7), 1306–1308. https://doi.org/10.1111/j.1532-5415.2009.02317.x

Heine, C., Browning, C. J., & Gong, C. H. (2019). Sensory Loss in China: Prevalence, Use of Aids, and Impacts on Social Participation. Frontiers in public health, 7, 5. https://doi.org/10.3389/fpubh.2019.00005

Henderson Sabes, J., & Sweetow, R. W. (2007). Variables predicting outcomes on listening and communication enhancement (LACE) training. International journal of audiology, 46(7), 374–383. https://doi.org/10.1080/14992020701297565

Hooper, R. (2014). Justifying sample size for a feasibility study: research design service. NIHR.

Informal Working Group on Prevention of Deafness and Hearing Impairment Programme Planning, & World Health Organization. (1991). Report of the Informal Working Group on Prevention of Deafness and Hearing Impairment Programme Planning (Programme for the Prevention of Deafness and Hearing Impairment, Issue. https://apps.who.int/iris/handle/10665/58839

Julious, S. A. (2005). Sample size of 12 per group rule of thumb for a pilot study. Pharm Stat, 4(4), 287-291. https://doi.org/10.1002/pst.185

Lam, L. C. W., Chau, R. C. M., Wong, B. M. L., Fung, A. W. T., Lui, V. W. C., & Tam, C. C. W. (2011). Interim follow-up of a randomized controlled trial comparing Chinese style mind body (Tai Chi) and stretching exercises on cognitive function in subjects at risk of progressive cognitive decline. Int J Geriatr Psychiatry 26(733-740).

Lawrence, B. J., Jayakody, D., Henshaw, H., Ferguson, M. A., Eikelboom, R. H., Loftus, A. M., & Friedland, P. L. (2018a). Auditory and Cognitive Training for Cognition in Adults with Hearing Loss: A Systematic Review and Meta-Analysis. Trends in hearing, 22, 2331216518792096. https://doi.org/https://doi.org/10.1177/2331216518792096

Lawrence, B. J., Jayakody, D., Henshaw, H., Ferguson, M. A., Eikelboom, R. H., Loftus, A. M., & Friedland, P. L. (2018b). Auditory and Cognitive Training for Cognition in Adults with Hearing Loss: A Systematic Review and Meta-Analysis. Trends in hearing, 22(2331216518792096). https://doi.org/10.1177/2331216518792096

Leung, A., Su, J. J., Lee, E., Fung, J., & Molassiotis, A. (2022). Intrinsic capacity of older people in the community using WHO Integrated Care for Older People (ICOPE) framework: a cross-sectional study. BMC Geriatrics, 22(1), 304. https://doi.org/10.1186/s12877-022-02980-1

Leung, G. T., de Jong Gierveld, J., & Lam, L. C. (2008). Validation of the Chinese translation of the 6-item De Jong Gierveld Loneliness Scale in elderly Chinese. Int Psychogeriatr, 20(6), 1262-1272. https://doi.org/10.1017/S1041610208007552

Lin, F. R., Ferrucci, L., An, Y., Goh, J. O., Doshi, J., & Metter, E. J. (2014). Association of hearing impairment with brain volume changes in older adults. Neuroimage, 90, 84-92. https://doi.org/10.1016/j.neuroimage.2013.12.059

Lin, F. R., Ferrucci, L., An, Y., Goh, J. O., Doshi, J., & Metter, E. J. (2014). Association of hearing impairment with brain volume changes in older adults. Neuroimage, 90, 84-92. https://doi.org/10.1016/j.neuroimage.2013.12.059

Lin, F. R., Ferrucci, L., Metter, E. J., An, Y., Zonderman, A. B., & Resnick, S. M. (2011). Hearing loss and cognition in the Baltimore Longitudinal Study of Aging. Neuropsychology, 25(6), 763–770. https://doi.org/10.1037/a0024238

Lipardo, D. S., & Tsang, W. (2018). Falls prevention through physical and cognitive training (falls PACT) in older adults with mild cognitive impairment: a randomized controlled trial protocol. BMC Geriatrics, 18(1), 193. https://doi.org/10.1186/s12877-018-0868-2

Livingston, G., Huntley, J., Sommerlad, A., Ames, D., Ballard, C., Banerjee, S., Brayne, C., Burns, A., Cohen-Mansfield, J., Cooper, C., Costafreda, S. G., Dias, A., Fox, N., Gitlin, L. N., Howard, R., Kales, H. C., Kivimäki, M., Larson, E. B., Ogunniyi, A., Orgeta, V., … Mukadam, N. (2020). Dementia prevention, intervention, and care: 2020 report of the Lancet Commission. Lancet (London, England), 396(10248), 413–446. https://doi.org/10.1016/S0140-6736(20)30367-6

Loughrey, D. G., Kelly, M. E., Kelley, G. A., Brennan, S., & Lawlor, B. A. (2018). Association of Age-Related Hearing Loss With Cognitive Function, Cognitive Impairment, and Dementia: A Systematic Review and Meta-analysis. JAMA otolaryngology-- head & neck surgery, 144(2), 115–126. https://doi.org/10.1001/jamaoto.2017.2513

Ludlow, K., Mumford, V., Makeham, M., Braithwaite, J., & Greenfield, D. (2018). The effects of hearing loss on person-centred care in residential aged care: a narrative review. Geriatric nursing (New York, N.Y.), 39(3), 296–302. <https://doi.org/>

Mamo, S. K., & Wheeler, K. A. (2021). The Combined Burden of Hearing Loss and Cognitive Impairment in a Group Care Setting for Older Adults. . Journal of speech, language, and hearing research: JSLHR, 64(2), 328–336. https://doi.org/10.1044/2020_JSLHR-20-00068

McIsaac, T. L., Lamberg, E. M., & Muratori, L. M. (2015). Building a framework for a dual task taxonomy. BioMed research international, 591475. https://doi.org/10.1155/2015/591475

Mewborn, C. M., Lindbergh, C. A., & Stephen Miller, L. (2017). Cognitive Interventions for Cognitively Healthy, Mildly Impaired, and Mixed Samples of Older Adults: A Systematic Review and Meta-Analysis of Randomized-Controlled Trials. Neuropsychology review, 27(4), 403–439. https://doi.org/10.1007/s11065-017-9350-8

Mick, P., Kawachi, I., & Lin, F. R. (2014). The association between hearing loss and social isolation in older adults. Otolaryngology--head and neck surgery: official journal of American Academy of Otolaryngology-Head and Neck Surgery, 150(3), 378–384. https://doi.org/10.1177/0194599813518021

Ng, J. H., & Loke, A. Y. (2015). Determinants of hearing-aid adoption and use among the elderly: a systematic review. International journal of audiology, 54(5), 291–300. https://doi.org/10.3109/14992027.2014.966922

Ng, J. H. & Loke, A. Y. (2019). Determinants of hearing aid adoption and use among Chinese elderly in Hong Kong. Dissertation. Hong Kong Polytechnic University: Hong Kong. https://theses.lib.polyu.edu.hk/handle/200/10429

Parial, L. L., Kor, P., Sumile, E. F., & Leung, A. (2022). Dual-task Zumba Gold for improving the cognition of people with mild cognitive impairment: A pilot randomized controlled trial. The Gerontologist, gnac081. https://doi.org/10.1093/geront/gnac081

Parial, L. L., Leung, A., Sumile, E. F., & Lam, S. C. (2021). Pilot testing of Dual-task Zumba Gold (DTZ) for community-dwelling people with mild cognitive impairment: A mixed-methods study. . Geriatric nursing (New York, N.Y.), 42(6), 1397–1407. https://doi.org/10.1016/j.gerinurse.2021.09.013

Reuter-Lorenz, P. A., & Park, D. C. (2014). How does it STAC up? Revisiting the scaffolding theory of aging and cognition. Neuropsychology review, 24(3), 355–370. https://doi.org/10.1007/s11065-014-9270-9

https://doi.org/10.1146/annurev.psych.59.103006.093656

Phirom, K., Kamnardsiri, T., & Sungkarat, S. (2020). Beneficial Effects of Interactive Physical-Cognitive Game-Based Training on Fall Risk and Cognitive Performance of Older Adults. International journal of environmental research and public health, 17(17), 6079. https://doi.org/10.3390/ijerph17176079

Prince, M., Wimo, A., Guerchet, M., Ali, G., Wu, Y., & Prina, M. (2015). The global impact of dementia: an analysis of prevalence, incidence, cost and trends. Retrieved 26 October 2022 from https://www.alz.co.uk/research/WorldAlzheimerReport2015.pdf

Reed, N. S., Altan, A., Deal, J. A., Yeh, C., Kravetz, A. D., Wallhagen, M., & Lin, F. R. (2019). Trends in Health Care Costs and Utilization Associated with Untreated Hearing Loss Over 10 Years. JAMA otolaryngology-- head & neck surgery, 145(1), 27-34. https://doi.org/10.1001/jamaoto.2018.2875

Rutherford, B. R., Brewster, K., Golub, J. S., Kim, A. H., & Roose, S. P. (2018). Sensation and Psychiatry: Linking Age-Related Hearing Loss to Late-Life Depression and Cognitive Decline. The American journal of psychiatry, 175(3), 215–224. https://doi.org/10.1176/appi.ajp.2017.17040423

Ryu, S. H. (2018). The Clinical Significance of Cognitive Interventions for the Patients with Mild Cognitive Impairment. J. Korean Neuropsychiatr. Assoc., 57, 23–29.

Saji, N., Makizako, H., Suzuki, H., Nakai, Y., Tabira, T., Obuchi, S., Kawai, H., Murotani, K., Katayama, N., Toba, K., Uchida, Y., & Nakashima, T. (2021). Hearing impairment is associated with cognitive function in community-dwelling older adults: A cross-sectional study. Archives of Gerontology and Geriatrics, 93(104302.). https://doi.org/10.1016/j.archger.2020.104302

Schwenk, M., Zieschang, T., Oster, P., & Hauer, K. (2010). Dual-task performances can be improved in patients with dementia: a randomized controlled trial. Neurology, 74(24), 1961–1968.

Shah, H., Albanese, E., Duggan, C., Rudan, I., Langa, K. M., Carrillo, M. C., & Dua, T. (2016). Research priorities to reduce the global burden of dementia by 2025. The Lancet Neurology, 15, 1285–1294. https://doi.org/10.1016/S1474- 4422(16)30235-6

Shukla, A., Harper, M., Pedersen, E., Goman, A., Suen, J. J., Price, C., Applebaum, J., Hoyer, M., Lin, F. R., & Reed, N. S. (2020b). Hearing Loss, Loneliness, and Social Isolation: A Systematic Review. Otolaryngology--head and neck surgery: official journal of American Academy of Otolaryngology-Head and Neck Surgery, 162(5), 622–633. https://doi.org/10.1177/0194599820910377

Stahl, S. M. (2017). Does treating hearing loss prevent or slow the progress of dementia? Hearing is not all in the ears, but who’s listening? CNS Spectr, 22(3), 247-250. https://doi.org/10.1017/S1092852917000268

Sweetow, R. W., & Henderson-Sabes, J. (2004). The case for LACE Listening and auditory communication enhancement training. The Hearing Journal, 57(3), 32-35, 38, 40. https://doi.org/10.1097/01.HJ.0000292371.26838.91

Sweetow, R. W., & Henderson-Sabes, J. (2006). The need for and development of an adaptive listening and communication enhancement (LACE™) program. J Am Acad Audiol 17, 538–558.

Uchida, Y., Nishita, Y., Tange, C., Sugiura, S., Otsuka, R., & Ueda, H. (2016). The longitudinal impact of hearing impairment on cognition differs according to cognitive domain

Front Aging Neurosci, 8 201. https://doi.org/10.3389/fnagi.2016.00201

Uchida, Y., Sugiura, S., Nishita, Y., Saji, N., Sone, M., & Ueda, H. (2019). Age-related hearing loss and cognitive decline - The potential mechanisms linking the two. Auris, nasus, larynx, 46(1), 1–9. https://doi.org/10.1016/j.anl.2018.08.010

Wayne, R. V., & Johnsrude, I. S. (2015). A review of causal mechanisms underlying the link between age-related hearing loss and cognitive decline. Ageing Res Rev, 23, 154-166. https://doi.org/10.1016/j.arr.2015.06.002

Wingfield, A., Tun, P. A., & McCoy, S. L. (2005). Hearing Loss in Older Adulthood: What It Is and How It Interacts with Cognitive Performance. Current Directions in Psychological Science., 14(3), 144-148. https://doi.org/10.1111/j.0963-7214.2005.00356.x

World Health Organisation. (2021). World Report on Hearing

Ye, X., Zhu, D., & He, P. (2021). The role of self-reported hearing status in the risk of hospitalisation among Chinese middle-aged and older adults. . International journal of audiology, 60(10), 754–761. https://doi.org/10.1080/14992027.2021.1871671

Yeo, B. S. Y., Song, H. J. J. M. D., Toh, E. M. S., Ng, L. S., Ho, C. S. H., Ho, R., Merchant, R. A., Tan, B. K. J., & Loh, W. S. (2022). Association of Hearing Aids and Cochlear Implants with Cognitive Decline and Dementia: A Systematic Review and Meta-analysis. JAMA neurology, 10.1001/jamaneurol.2022.4427. Advance online publication. https://doi.org/10.1001/jamaneurol.2022.4427

Yeung, P. Y., Wong, L. L., Chan, C. C., Leung, J. L., & Yung, C. Y. (2014). A validation study of the Hong Kong version of Montreal Cognitive Assessment (HK-MoCA) in Chinese older adults in Hong Kong. Hong Kong medical journal = Xianggang yi xue za zhi, 20(6), 504–510. https://doi.org/10.12809/hkmj144219

Yu, R., Leung, G., & Woo, J. (2021). Randomized Controlled Trial on the Effects of a Combined Intervention of Computerized Cognitive Training Preceded by Physical Exercise for Improving Frailty Status and Cognitive Function in Older Adults. International journal of environmental research and public health, 18(4), 1396. https://doi.org/10.3390/ijerph18041396

1. *Key words*

Please provide up to **4 key words** for your project:

| First | Hearing loss | Second | Cognitive impairment |
| --- | --- | --- | --- |
|  |  |  |  |
| Third | Dual-task | Fourth | Older adults |

1. Duration of Project

Duration: ____24_________________ months

| Start Date: | 3/4/2023 | Expected Completion Date: | 2/4/2025 |
| --- | --- | --- | --- |
