# Supplementary material for: Web-Based Gamified Auditory-Cognitive Dual-Task Training for Older Adults With Age-Related Hearing Loss: Pilot Randomized Controlled Trial
Source: JMIR Aging. 2026 Jun 16;9:e84083. doi: 10.2196/84083 (PMC13271602; doi:10.2196/84083)
Supplement: Multimedia Appendix 2 [file aging-v9-e84083-s002.pdf]

**Supplement File 3. A Log for Checking Study Feasiblity**

| Subjec | Group (H, | Age (60 or a | Hearing ability by PTA | MoCA score | Included/w | Reason of no included | signed ICF | Demographic information and T0 | Start date | Week 6 | completion date | Week 12 | First appointment | Second appointment | Third appointment |
|---|---|---|---|---|---|---|---|---|---|---|---|---|---|---|---|
| 1 | W | 74 | mild | 30 | Included | | Yes | Yes | 20241030 | 20241203 | 20250114 | Yes | 20241030 14:00 | 20241203 10:00 | 20250114 15:00 |
| 2 | H | 71 | mild to moderate | 28 | Included | | Yes | Yes | 20240821 | 20241009 | 20241126 | Yes | By CFSC | By CFSC | By CFSC |
| 3 | W | 61 | mild | 23 | Included | | Yes | Yes | 20241107 | 20241212 | 20250206 | Yes | 20241107 18:15 | 20241212 17:45 | 20250206 18:30 |
| 4 | H | 70 | mild to moderate | 28 | Included | | Yes | Yes | 20240821 | 20241010 | 20241126 | Yes | By CFSC | By CFSC | By CFSC |
| 5 | H | 69 | mild to moderate | 21 | Included | | Yes | Yes | 20240821 | 20241017 | 20241209 | Yes | By CFSC | By CFSC | By CFSC |
| 6 | H | 62 | mild | 24 | Included | | Yes | Yes | 20240823 | 20241017 | 20241126 | Yes | By CFSC | By CFSC | By CFSC |
| 7 | W | 66 | mild | 23 | Included | | Yes | Yes | 20241107 | 20241212 | 20250206 | Yes | 20241107 18:15 | 20241212 17:45 | 20250206 18:30 |
| 8 | W | 66 | mild | 29 | Included | | Yes | Yes | 20241111 | 20241218 | 20250207 | Yes | 20241111 14:30 | 20241218 14:45 | 20250207 10:00 |
| ~~8~~ | W | | normal | | Withdraw | No hearing loss | Yes | No | | | | | 20241030 10:30 | | |
| 9 | W | 61 | mild | 27 | Included | | Yes | Yes | 20241025 | 20241206 | 20250116 | Yes | 20241025 10:30 | 20241206 10:00 | 20250116 10:00 |
| ~~10~~ | H | 65 | moderate | 27 | Withdraw | | Yes | Yes | | | | | By CFSC | | |
| 10 | H | 73 | mild | 25 | Included | | Yes | Yes | 20250218 | 20250407 | 20250528 | Yes | 20240218 11:00 | 20250407 14:00 | 20250528 14:00 |
| | | | | | Withdraw | lost contact | Yes | No | | | | | | | |
| | | | | | Withdraw | Eye problems and no time | Yes | No | | | | | | | |
| 11 | W | 61 | mild | 26 | Included | | Yes | Yes | 20241028 | 20241202 | 20250114 | Yes | 20241028 14:00 | 20241202 15:45 | 20250114 10:00 |
| 12 | W | 67 | mild | 30 | Included | | Yes | Yes | 20241108 | 20241209 | 20250120 | Yes | 20241108 10:30 | 20241209 10:45 | 20250120 18:30 |
| ~~12~~ | ~~W~~ | | normal | | Withdraw | No hearing loss | Yes | Yes | | | | | 20241028 15:00 | | |
| 13 | H | 72 | Right: mild; Left: moderate to severe | 27 | Included | | Yes | Yes | 20241022 | 20241203 | 20250212 | Yes | 20241022 14:00 | 20241203 14:00 | 20250212 14:00 |
| 14 | H | 65 | mild | 29 | Included | | Yes | Yes | 20241025 | 20241203 | 20250114 | Yes | 20241025 14:00 | 20241203 11:30 | 20250114 11:00 |
| 15 | W | 64 | mild | 24 | Included | | Yes | Yes | 20241028 | 20241202 | 20250113 | Yes | 20241028 10:30 | 20241202 10:45 | 20250113 15:00 |
| 16 | H | 70 | moderate | 27 | Included | | Yes | Yes | 20241024 | 20241204 | 20250207 | Yes | 20241024 10:30 | 20241204 14:00 | 20250203 11:00 |
| 17 | H | 74 | mild | 28 | Included | | Yes | Yes | 20241104 | 20241217 | 20250211 | Yes | 20241104 15:30 | 20241217 14:00 | 20250211 14:00 |
| 18 | H | 64 | right: mild; left: severe | 29 | Included | | Yes | Yes | 20241118 | 20241231 | 20250124 | Yes | 20241118 11:00 | 20241231 14:00 | 20250124 14:00 |
| 19 | W | 60 | mild | 30 | Included | | Yes | Yes | 20241028 | 20241206 | 20250115 | Yes | 20241028 17:45 | 20241206 20:00 | 20250115 19:30 |
| 20 | W | 64 | mild | 26 | Included | | Yes | Yes | 20241030 | 20241202 | 20250113 | Yes | 20241030 15:30 | 20241202 14:00 | 20250113 11:00 |
| 21 | W | 70 | moderate | 25 | Included | | Yes | Yes | 20241029 | 20241203 | 20250116 | Yes | 20241029 10:30 | 20241203 10:45 | 20250116 11:00 |
| 22 | H | 68 | Right: moderate; Left: mild | 30 | Included | | Yes | Yes | 20241025 | 20241204 | 20250206 | Yes | 20241025 15:30 | 20241204 16:15 | 20250206 12:00 |
| ~~23~~ | H | | | | Withdraw | 20241203 indicates withdrawal | No | | | | | | | | |
| 23 | H | 61 | mild | 28 | Included | | Yes | Yes | 20241204 | 20250120 | 20250306 | Yes | 20241204 11:30 | 20250120 16:00 | 20250306 11:00 |
| 24 | H | 68 | mild | 29 | Included | | Yes | Yes | 20241204 | 20250115 | 20250217 | Yes | 20241204 14:45 | 20250115 14:00 | 20250217 15:00 |
| 25 | W | | | | Withdraw | 20241206 withdraw due to recent serious injury, cannot physically visit | No | No | | | | | | | |
| 25 | W | 62 | mild | 27 | Included | | Yes | Yes | 20241209 | 20250113 | 20250224 | Yes | 20241209 19:30 | 20250113 19:30 | 20250224 19:15 |
| 26 | W | 71 | mild | 23 | Included | | Yes | Yes | 20241111 | 20241219 | 20250206 | Yes | 20241111 16:00 | 20241219 10:45 | 20250206 11:00 |
| 27 | W | 61 | left:normal; right: mild | 30 | Included | | Yes | Yes | 20241104 | 20241206 | 20250120 | Yes | 20241104 13:00 | 20241206 10:45 | 20250120 10:00 |
| 28 | W | 77 | moderate | 25 | Included | | Yes | Yes | 20241101 | 20241213 | 20250124 | Yes | 20241101 14:00 | 20241214 15:00 | 20250125 11:00 |
| 29 | W | 73 | mild | 30 | Included | | Yes | Yes | 20241104 | 20241210 | 20250120 | Yes | 20241104 11:00 | 20241210 10:00 | 20250120 15:00 |
| 30 | W | 69 | mild | 23 | Included | | Yes | Yes | 20241101 | 20241207 | 20250117 | Yes | 20241101 14:00 | 20241208 10:00 | 20250118 16:00 |
| 31 | H | 68 | mild | 26 | Included | | Yes | Yes | 20241108 | 20241211 | 20250210 | Yes | 20241108 15:30 | 20241211 11:30 | 20250210 14:00 |
| 32 | W | 74 | right:moderate; left:mild | 30 | Included | | Yes | Yes | 20241107 | 20241230 | 20250303 | Yes | 20241107 16:00 | 20241230 14:45 | 20250303 10:00 |
| 33 | W | 73 | mild | 29 | Included | | Yes | Yes | 20241105 | 20241216 | 20250128 | Yes | 20241105 10:30 | 20241216 10:45 | 20250128 11:00 |
| 34 | H | 68 | mild | 27 | Included | | Yes | Yes | 20241106 | 20241212 | 20250204 | Yes | 20241106 10:30 | 20241212 10:00 | 20250204 14:00 |
| 35 | H | 60 | mild | 30 | Included | | Yes | Yes | 20241106 | 20241211 | 20250120 | Yes | 20241106 14:00 | 20241211 10:45 | 20250120 14:00 |
| 36 | W | 68 | mild | 24 | Included | | Yes | Yes | 20241106 | 20241209 | 20250122 | Yes | 20241106 15:30 | 20241209 11:30 | 20250122 15:00 |
| 37 | H | 76 | left:profound; right:mild | 28 | Included | | Yes | Yes | 20241111 | 20241217 | 20250204 | Yes | 20241111 11:00 | 20241217 15:30 | 20250204 15:00 |
| 38 | H | 61 | mild | 23 | Included | | Yes | Yes | 20241108 | 20241210 | 20250113 | Yes | 20241108 14:00 | 20241210 14:00 | 20250113 12:00 |
| 39 | W | 69 | right:moderate | 30 | Included | | Yes | Yes | 20241113 | 20241220 | 20250207 | Yes | 20241113 14:00 | 20241220 10:45 | 20250207 16:00 |
| 40 | W | 60 | mild | 24 | Included | | Yes | Yes | 20241204 | 20250117 | 20250227 | Yes | 20241204 18:15 | 20250117 18:15 | 20250227 18:15 |
| 41 | W | 71 | mild | 25 | Included | | Yes | Yes | 20241112 | 20241216 | 20250206 | Yes | 20241112 10:30 | 20241216 14:00 | 20250206 15:00 |
| 42 | H | 70 | moderate | 29 | Included | | Yes | Yes | 20241114 | 20241219 | 20250204 | Yes | 20241114 14:30 | 20241219 10:00 | 20250204 16:00 |
| 43 | H | 68 | left:mild; right:moderate | 26 | Included | | Yes | Yes | 20241113 | 20241218 | 20250127 | Yes | 20241113 15:30 | 20241218 10:00 | 20250127 11:00 |
| ~~44~~ | H | 63 | mild | 30 | Withdraw | lost contact | Yes | No | | | | | | | |
| 44 | H | 72 | mild | 28 | Included | | Yes | Yes | 20250218 | 20250407 | 20250528 | Yes | 20240218 11:00 | 20250407 14:00 | 20250528 11:00 |
| 45 | H | 68 | moderate | 26 | Included | | Yes | Yes | 20241114 | 20241218 | 20250211 | Yes | 20241114 16:30 | 20241218 10:45 | 20250211 11:00 |
| 46 | W | 68 | mild | 30 | Included | | Yes | Yes | 20241115 | 20241220 | 20250207 | Yes | 20241115 10:30 | 20241220 10:00 | 20250204 11:00 |
| 47 | W | 69 | left:moderate; right:mild | 27 | Included | | Yes | Yes | 20241118 | 20250102 | 20250210 | Yes | 20241118 14:00 | 20250102 14:00 | 20250210 15:00 |
| 48 | W | 66 | moderate | 24 | Included | | Yes | Yes | 20241118 | 20241230 | 20250212 | Yes | 20241118 15:30 | 20241230 14:00 | 20250212 15:00 |
| 49 | H | 65 | moderate | 28 | Included | | Yes | Yes | 20241113 | 20241217 | 20250203 | Yes | 20241113 11:00 | 20241217 10:00 | 20250203 11:00 |
| 50 | H | 73 | mild | 30 | Included | | Yes | Yes | 20241119 | 20241230 | 20250205 | Yes | 20241119 16:00 | 20241230 16:15 | 20250205 11:00 |
| 51 | W | 64 | mild | 28 | Included | | Yes | Yes | 20241119 | 20250103 | 20250214 | Yes | 20241119 10:30 | 20250103 16:15 | 20250214 15:00 |
| ~~52~~ | W | 74 | left:moderate; right:profound | 18 | Withdraw | No hearing loss | Yes | No | | | | | | | |
| 52 | W | 79 | mild | 25 | Included | | Yes | Yes | 20250212 | 20250408 | 20250616 | Yes | 20250212 11:00 | 20240409 19:30 | 20250213 15:00 |
| 53 | H | 72 | mild | 25 | Included | | Yes | Yes | 20241122 | 20241230 | 20250214 | Yes | 20241122 12:00 | 20241230 10:45 | 20250214 11:00 |
| 54 | W | 70 | moderate | 21 | Included | | Yes | Yes | 20241120 | 20250102 | 20250212 | Yes | 20241120 13:00 | 20250102 10:45 | 20250212 16:00 |
| 55 | H | 66 | moderate | 30 | Included | | Yes | Yes | 20241121 | 20250117 | 20250303 | Yes | 20241121 15:30 | 20250117 16:30 | 20250303 18:00 |
| 56 | H | 61 | mild | 27 | Included | | Yes | Yes | 20241121 | 20250108 | 20250205 | Yes | 20241121 10:30 | 20250108 14:00 | 20250205 12:00 |
| 57 | H | 63 | mild | 25 | Included | | Yes | Yes | 20241120 | 20250108 | 20250207 | Yes | 20241120 10:30 | 20250108 11:30 | 20250207 11:00 |
| 58 | W | 69 | mild | 28 | Included | | Yes | Yes | 20241122 | 20241230 | 20250210 | Yes | 20241122 10:30 | 20241230 11:30 | 20250210 11:00 |
| 59 | H | 66 | left:mild; right:moderate | 27 | Included | | Yes | Yes | 20241206 | 20250113 | 20250217 | Yes | 20241206 14:00 | 20250113 16:00 | 20250217 11:00 |
| 60 | H | 66 | mild | 28 | Included | | Yes | Yes | 20241118 | 20250120 | 20250306 | Yes | 20241118 10:00 | 20250120 11:00 | 20250306 14:00 |
